# Microhabitat Governs the Microbiota of the Pinewood Nematode and Its Vector Beetle: Implication for the Prevalence of Pine Wilt Disease

**DOI:** 10.1128/spectrum.00783-22

**Published:** 2022-06-27

**Authors:** Haokai Tian, Lilin Zhao, Tuuli-Marjaana Koski, Jianghua Sun

**Affiliations:** a State Key Laboratory of Integrated Management of Pest Insects and Rodents, Institute of Zoology, Chinese Academy of Sciences, Beijing, China; b College of Life Science, Institute of Life Science and Green Development, Hebei University, Baoding, China; c CAS Center for Excellence in Biotic Interactions, University of Chinese Academy of Sciences, Beijing, China; South China Agricultural University

**Keywords:** bacterial and fungal community, community assembly, microhabitat, microbial transmission, plant-nematode-vector beetle, pinewood nematode

## Abstract

Our understanding of environmental acquisition of microbes and migration-related alteration of microbiota across habitats has rapidly increased. However, in complex life cycles, such as for many parasites, exactly how these microbes are transmitted across multiple environments, such as hosts and habitats, is unknown. Pinewood nematode, the causal agent of the globally devastating pine wilt disease, provides an ideal model to study the role of microbiota in multispecies interactions because its successful host invasion depends on the interactions among its vector insects, pine hosts, and associated microbes. Here, we studied the role of bacterial and fungal communities involved in the nematode’s life cycle across different micro- (pupal chamber, vector beetle, and dispersal nematodes) and macrohabitats (geographical locations). We identified the potential sources, selection processes, and keystone taxa involved in the host pine-nematode-vector beetle microbiota interactions. Nearly 50% of the microbiota in vector beetle tracheae and ~60% that of third-stage dispersal juveniles were derived from the host pine (pupal chambers), whereas 90% of bacteria of fourth-stage dispersal juveniles originated from vector beetle tracheae. Our results also suggest that vector beetles’ tracheae selectively acquire some key taxa from the microbial community of the pupal chambers. These taxa will be then enriched in the dispersal nematodes traveling in the tracheae and hence likely transported to new host trees. Taken together, our findings contribute to the critical information toward a better understanding of the role of microbiota in pine wilt disease, therefore aiding the knowledge for the development of future biological control agents.

**IMPORTANCE** Our understanding of animal microbiota acquisition and dispersal-mediated variation has rapidly increased. In this study, using the model of host pine-pinewood nematode-vector beetle (*Monochamus* sp.) complex, we disentangled the routes of microbial community assembly and transmission mechanisms among these different participants responsible for highly destructive pine wilt disease. We provide evidence that the microhabitat is the driving force shaping the microbial community of these participants. The microbiota of third-stage dispersal juveniles (L_III_) of the nematodes collected around pupal chambers and of vector beetles were mainly derived from the host pine (pupal chambers), whereas the vector-entering fourth-stage dispersal juveniles (L_IV_) of the nematodes had the simplest microbiota community, not influencing vector’s microbiota. These findings enhanced our understanding of the variation in the microbiota of plants and animals and shed light on microbiota acquisition in complex life cycles.

## INTRODUCTION

Understanding the processes of how plants and animals acquire their microbiomes has the potential to improve ecosystem restoration, agricultural sustainability, and disease management (1, 2). Disentangling the ecological processes governing microbial community assembly and their relative impacts has proven challenging. Therefore, attempts to understand and manipulate microbiota should not only focus on an individual host but also investigate the microbiota communities in its surrounding environment (3). Increasing numbers of studies have focused on the microbial community assembly and transmission, for example, from mother to offspring (4, 5), as well as in soil-plant-insect (6, 7), aquatic (8, 9), and trophic network (10, 11) systems. Although recent studies continue to reveal the role of the environment as one of the key factors shaping the microbial community of organisms (12–15), the extent to which and how microbes are spread across different microbial hosts or habitats are elusive. For example, investigation of microbiota acquisition from plants to leaf-feeding herbivores and their predators has shown greater similarity in the microbial community among adjacent trophic levels than in the more distant ones (10, 16). However, the transmission of microbiota from lower to higher trophic levels is not always straightforward, as demonstrated by the results finding by greater similarity in the microbiota composition of leaf-chewing insects to its host plant’s soil compared to the microbiota in the host plant (6). Therefore, identifying the transmission route how certain microbes are spread across hosts and habitats warrants further research (1). Microbiota transmission especially in multispecies-level interactions, such as in systems where parasitic organisms rely on animal vectors to reach their final hosts, is unknown. Understanding microbiota transmission and assembly in such systems would not only improve our understanding of interspecific microbiota acquisition but also aid the development of biocontrol methods against many plant and animal parasites.

**FIG 1:**
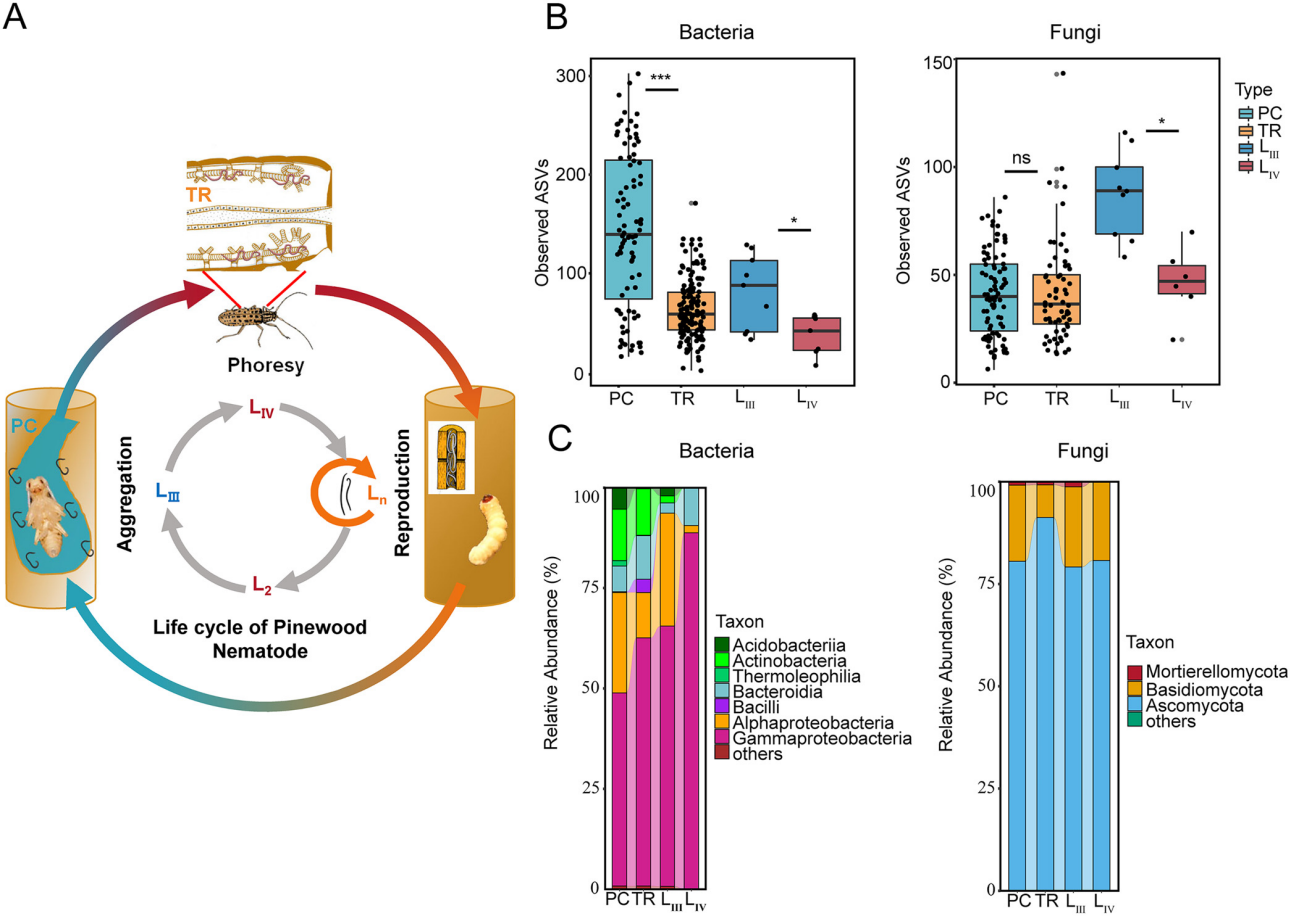
Diversity and composition of bacterial and fungal communities in four pinewood nematode-associated microhabitats. (A) Schematic presentation of the complex life cycle of pinewood nematode. The gray circle represents different larval stages and colored arrows represent the different habitats of nematodes. (B) Microbial alpha diversity across all five sites in pupal chamber, tracheae, L_III_, and L_IV_ samples based on the observed ASVs. (C) Composition of bacterial classes and fungal phylum at different microhabitats. Kruskal-Wallis with FDR adjusted, *, *P* < 0.05; **, *P* < 0.01; ***, *P* < 0.001. PC, pupal chamber; TR, trachea; L_III_, third-stage dispersal juveniles; L_IV_, fourth-stage dispersal juveniles.

The pinewood nematode (PWN), *Bursaphelenchus xylophilus* (Steiner & Buhrer) Nickle, is an example of such parasitic organisms, being a global quarantine invasive pest and the causal agent of pine wilt disease (PWD). Native to North America, pinewood nematode has invaded and caused severe damage in Japan, China, and South Korea, as well as in some parts of Europe, and now poses a serious threat to pine forests globally (17–19). In China, despite tremendous prevention and control efforts implemented over the past 40 years, pinewood nematode has spread from its initial southern range also to the high-latitude range in the northern part of the country (20–22). Compared to free-living entomophilic or entomopathogenic nematodes, pinewood nematode has a unique life cycle across a range of different microhabitats. The nematode’s life cycle consists of a propagative (adult and four larval stages, L_1_ to L_4_) and a dispersal life stages (dispersal juveniles, L_III_ and L_IV_), each life stage having a distinct relationship with the main host (pine tree) and vector beetle. During the summer, pinewood nematodes are vectored to healthy host pine trees by the vector beetles (*Monochamus* sp.). If the conditions inside the host pine are favorable, the nematodes rapidly develop into the reproductive adult stages through four propagative larval stages (L_1_ to _4_). The nematodes often attain high population density, leading to the death of the host pine. However, under unfavorable conditions (usually in winter), pinewood nematodes enter dispersal stage and molt from second-stage propagative larvae (L_2_) to third-stage dispersal juveniles (L_III_) (23). During the following spring, third-stage dispersal juveniles start to aggregate around the pupal chambers of vector beetles attracted by the volatiles released by beetle larvae (22, 24). Once the beetle larvae have reached late pupal stages, third-stage dispersal juveniles molt to fourth-stage dispersal juveniles (L_IV_). These nematodes then enter the spiracles of adult vector beetles to reach their tracheal system, in which pinewood nematodes are transported to new healthy host pines, where they start a new life cycle (Fig. 1A) (25, 26). This system provides an ideal model to study interspecies microbiota transmission because the transmission of pine wilt disease and associated microbiota requires multiple interactions among host pine, pinewood nematode, and the vector beetle.

Recent studies investigating the associations among the bacterial communities of pinewood nematode, host pine, and vector beetle suggest that microbes play a nonnegligible role in the prevalence and pathogenicity of pine wilt disease (27). The focus of most previous studies has been on investigating bacterial isolates of the nematodes, host pine and vector beetle and identifying their role of being either beneficial to or nematocidal against pinewood nematode (22, 27, 28). However, those studies focused more on certain microbial species and hence are unable to present a full picture of the microbial community in the pine wilt disease complex (29–32). Another knowledge gap lies in the understanding the role of microbiota in multispecies interactions among pinewood nematode, host pine, and vector beetle in the initiation and progress of pine wilt disease. The few studies on this topic include association analyses between bacterial communities of different combinations of nematodes, infected trees, soil environments, and various instars of vector beetles (33–35). The pupal chamber in host pines is a shared environment for pinewood nematode and its vector beetle and has been shown to harbor enormous diversity of bacteria compared to healthy pines (28, 34, 36). However, the question how exactly microbiota is assembled and transferred within the pine wilt disease complex, for example, from pupal chamber to the vector beetle’s tracheae, or from one host tree to another, is still unanswered. Furthermore, only a few studies have investigated the fungal communities involved in different stages of pinewood nematode’s life cycle, despite the fact that those fungi have been shown to be the most common and best-studied associates of bark beetles and are beneficial for the nematode-beetle complex (35, 37). However, some studies indicate that entomophilic nematodes do not carry their own bacterial communities during host infestations (38), making it therefore possible that pinewood nematode’s microbiota is solely acquired from host tree or insect vector. Because pine wilt disease is a result of an assemblage of pinewood nematode, vector beetle, host pine, and their associated microbiota, understanding the role of microbiota in the initiation and progress of pine wilt disease requires revealing the routes of microbial community assembly and transmission mechanisms among the host pine-pinewood nematode-vector beetle complex.

In this study, we hypothesized that the microbiota of the pine wilt disease complex will be gradually filtered down from the host pine (pupal chambers) toward vector beetles’ tracheae, being lowest in pinewood nematode. More specifically, we examined bacterial and fungal communities in pupal chambers, beetle tracheae, and two dispersal juvenile stages of pinewood nematode (L_III_ and L_IV_) in five different geographical locations. We aimed to assess: (i) how micro- and macrohabitats interactively shape the microbiota assembly and co-occurrence patterns in this unique complex life cycle and (ii) to identify the potential sources and keystone taxa in the microbial community of the symbiotic nematode-beetle disease complex for potential manipulation in management and control of pine wilt disease.

## RESULTS

### Microbiota diversity in different pinewood nematode micro- and macrohabitats.

We analyzed the microbiota (bacteria and fungi) associated with four microhabitat participants involved in the pinewood nematode life cycle: host pine (pupal chamber), vector beetles (tracheae), and two dispersal stages of pinewood nematodes juveniles (Fig. 1A; see also Tables S1 and S2 in the supplemental material for all samples collected in this study) and found that microbiota varied across these different microhabitats. Amplicon sequence variant (ASV) richness and diversity of bacteria and fungi were significantly decreased in fourth-stage dispersal juveniles compared to third-stage dispersal juveniles (Fig. 1B, Fig. S1; Kruskal-Wallis with FDR adjusted, *P* < 0.05). According to the results of nonmetric multidimensional scaling (NMDS) ordinations, the variations of bacterial and fungal community structures among different years and sites are mainly explained by sample types (Fig. S2). In general, the bacterial and fungal compositions in all these investigated microhabitats were similar. At the class level, *Gammaproteobacteria* (48.1%) and *Alphaproteobacteria* (25.0%) were the most abundant classes of bacteria, while Ascomycota (80.5%) and Basidiomycota (18.6%) were the most abundant phyla for fungi among the four microhabitats (Fig. 1C).

Among macrohabitats, the northern sites in Liaoning (LN) had the highest population density of pinewood nematodes, while the southern sites in Anhui (AH), Zhejiang (ZJ), and Jiangsu (JS), had very low nematode densities (Fig. S3A and B). According to the random forest model, the most important predictor for the Shannon index of bacterial microbiota in pupal chambers was the number of vector beetles, whereas for fungi, the number of pinewood nematodes was the most important predictor (Fig. S3C, D: bacteria: *R*^2^ = 54.81; fungi: *R*^2^ = 52.8, Table S4). Site was the dominating factor explaining variation in microbial community composition of pupal chambers (PERMANOVA: bacteria: *R*^2^ = 0.220, *P* = 0.01; fungi: *R*^2^ = 0.426, *P* = 0.01). In addition, among-site variation affected pupal chambers and tracheae-associated microbial communities more strongly than temporal variation (Fig. S3E, F).

### Linkages of microbiota inhabiting pupal chamber and trachea.

For fungi, the Shannon index for pupal chambers was lower than for tracheae in all five sites, whereas the opposite was true for bacteria, the diversity being higher in pupal chambers compared to tracheae (Wilcoxon rank sum test, *P* < 0.05). For both bacteria and fungi, the community structure in pupal chambers and tracheae differed significantly in all sites (ANOSIM: bacteria: *R *= 0.60, *P* = 0.001; fungi: *R *= 0.67, *P* = 0.001) (Fig. S4C, D).

**FIG 2:**
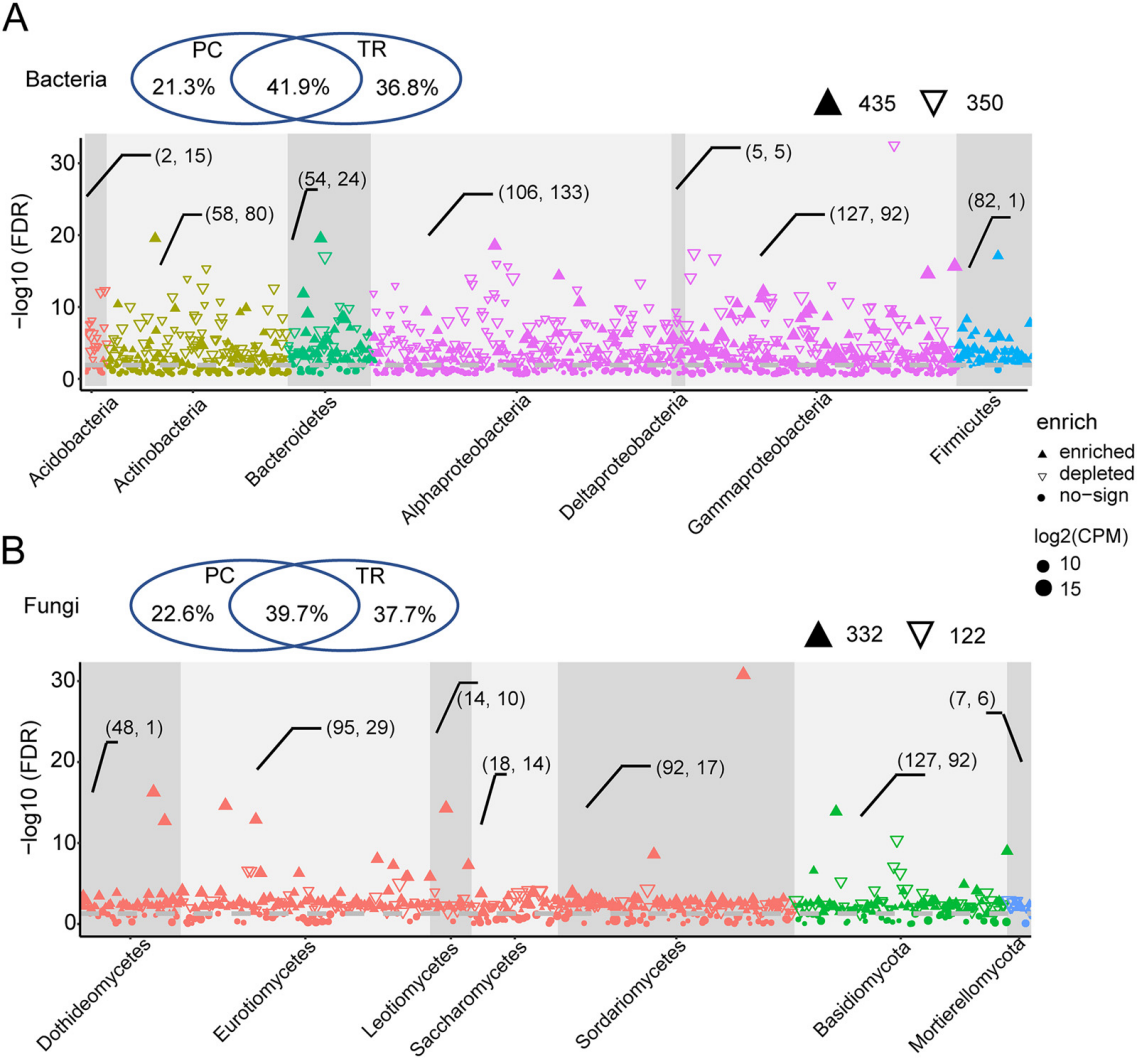
Taxonomic classification of different bacteria and fungi between pupal chambers and tracheae. The Manhattan plot shows ASVs enriched in tracheae samples for bacteria (A) and fungi (B). Each dot or triangle represents a single ASV. Venn diagrams above show the shared and unique ASVs between pupal chambers and tracheae. ASVs enriched or depleted in tracheae are represented by filled or empty triangles, respectively (FDR adjusted *P* < 0.05). ASVs are grouped by taxonomic order and colored according to the phylum (class for Ascomycota and Proteobacteria). “(2, 15)” means the number of enriched (2) and depleted (15) ASVs. PC, pupal chamber; TR, trachea; CPM, counts per million.

Despite these differences, pupal chambers and tracheae shared many of the ASVs and thus had very similar microbiota compositions. For example, the shared ASVs accounted for 41.9% (877) and 39.7% (332) of total bacterial and fungal ASVs, respectively (Fig. 2A and B; Fig. S4A, B). ASVs enriched in tracheae belonged to a wide range of bacterial and fungal phyla. Among these ASVs, 435 and 332 were enriched, 350 and 122 were depleted compared to pupal chambers, whereas 308 and 141 had no significant change in bacterial and fungal communities of tracheae compared to pupal chambers (Fig. 2A, B). The highest number of enriched ASVs in tracheae belonged to Gammaproteobacteria (127) and Eurotiomycetes (95). For bacterial taxa, the top four families, *Burkholderiaceae*, *Enterobacteriaceae*, *Pseudomonadaceae*, and *Nocardiaceae*, were enriched in tracheae, whereas families *Rhodanobacteraceae*, *Spingomonadaceae*, and *Acetobacteraceae* were depleted. For fungal taxa, *Ophiostomataceae*, *Hyphodermataceae*, and *Pichiaceae* were depleted in tracheae, whereas Aspergillaceae and some families with abundance of <5% were enriched in tracheae (Fig. S5A, B). When comparing tracheae samples from *M. alternatus* with and without fourth-stage dispersal juveniles from the same host pine species and similar latitude from three sites (AH, ZJ, and JS), no significant differences in microbiota diversity or composition were observed (*P* > 0.05, Fig. S6).

### Microbial community assembly processes across different pinewood nematode macrohabitats.

**FIG 3:**
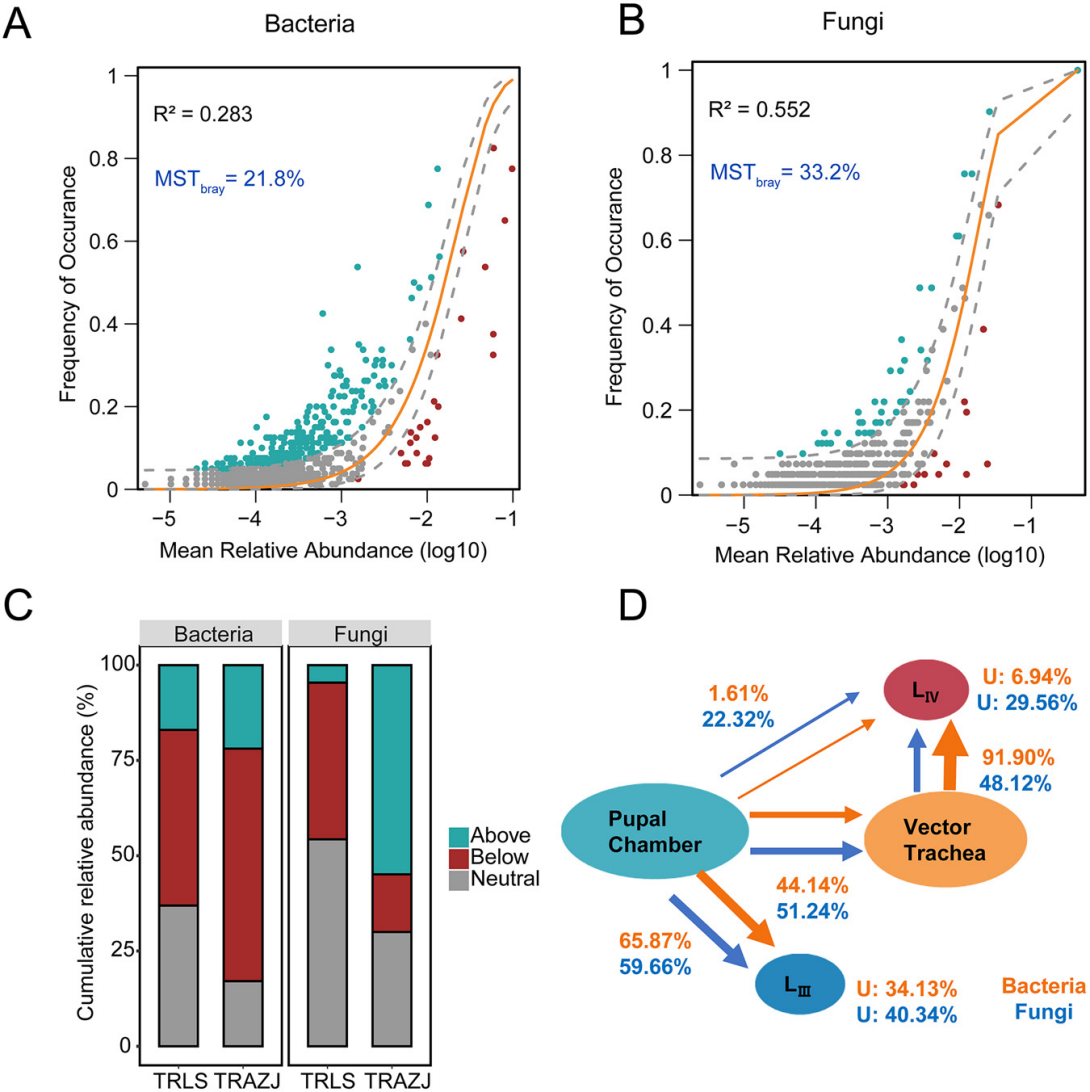
Microbial community assembly processes of the pinewood nematode life cycle. Shown is the Sloan neutral model for bacterial (A) and fungal (B) communities for tracheae samples across three southern sites. Gray dashed lines represent 95% confidence intervals around the model prediction and the ASVs fitting the model are colored gray. The ASVs occurring more frequently than predicted by the model are colored blue, and ASVs that occur less frequently than predicted are colored red. Letters in blue show the MST value based on Bray-Curtis distances. (C) The cumulative relative abundance distribution of three types of ASVs of the neutral model in northern (TRLS) and southern (TRAZJ) sites. (D) Source route of host microbiota showing the potential source of bacterial and fungal communities for different host types involved in the pinewood nematode life cycle. U, unknown source; TRLS, trachea samples from Liaoning and Shaanxi; TRAZJ, trachea samples from Anhui, Zhejiang, and Jiangsu.

When the data sets from northern and southern sites of trachea samples were analyzed separately (assuming that the tracheae samples represent a metacommunity within each sampling location), the *R*^2^ of the fungal community was higher than in the bacterial community (for fungi, *R*^2^ = 0.410 and 0.552, and for bacteria, *R*^2^ = 0.160 and 0.283 in northern and southern sites, respectively) (Fig. 3A and B; Fig. S7A, B). The modified stochasticity ratio (MST) value suggests that the microbial community in tracheae at all sites was more strongly driven by deterministic assembly processes (MST < 50%). For bacteria, the cumulative relative abundances of the ASVs below prediction were high in both northern and southern sites (46.1% and 61.0%, respectively) and were dominated by Gammaproteobacteria (47.4% in TRAZJ; 40.3% in TRLS). For fungi, the cumulative relative abundance of the ASVs above the predicted value in tracheae was higher in samples from southern sites (54.9%) compared to the northern sites (4.6%) and were dominated by Sordariomycetes (45.5% in TRAZJ) (Fig. 3C, Fig. S8, Table S5).

FEAST analysis revealed that different proportion of microbes in the tracheae and dispersal nematodes had a plausible origin from the pupal chambers microbiota (Fig. 3D). Overall, 44.13% of bacterial and 51.24% of fungal communities in tracheae samples were originated from pupal chambers, whereas a large proportion of microbes in the third-stage dispersal juveniles (65.87% and 59.66% for bacteria and fungi, respectively) had a plausible origin from pupal chambers. Surprisingly, a higher proportion of microbes in fourth-stage dispersal juveniles had a plausible origin from tracheae compared to pupal chambers (bacteria and fungi derived from tracheae: 91.90% and 48.12%, respectively) (Fig. 3D).

### Keystone taxa across the pinewood nematode’s life cycle.

**FIG 4:**
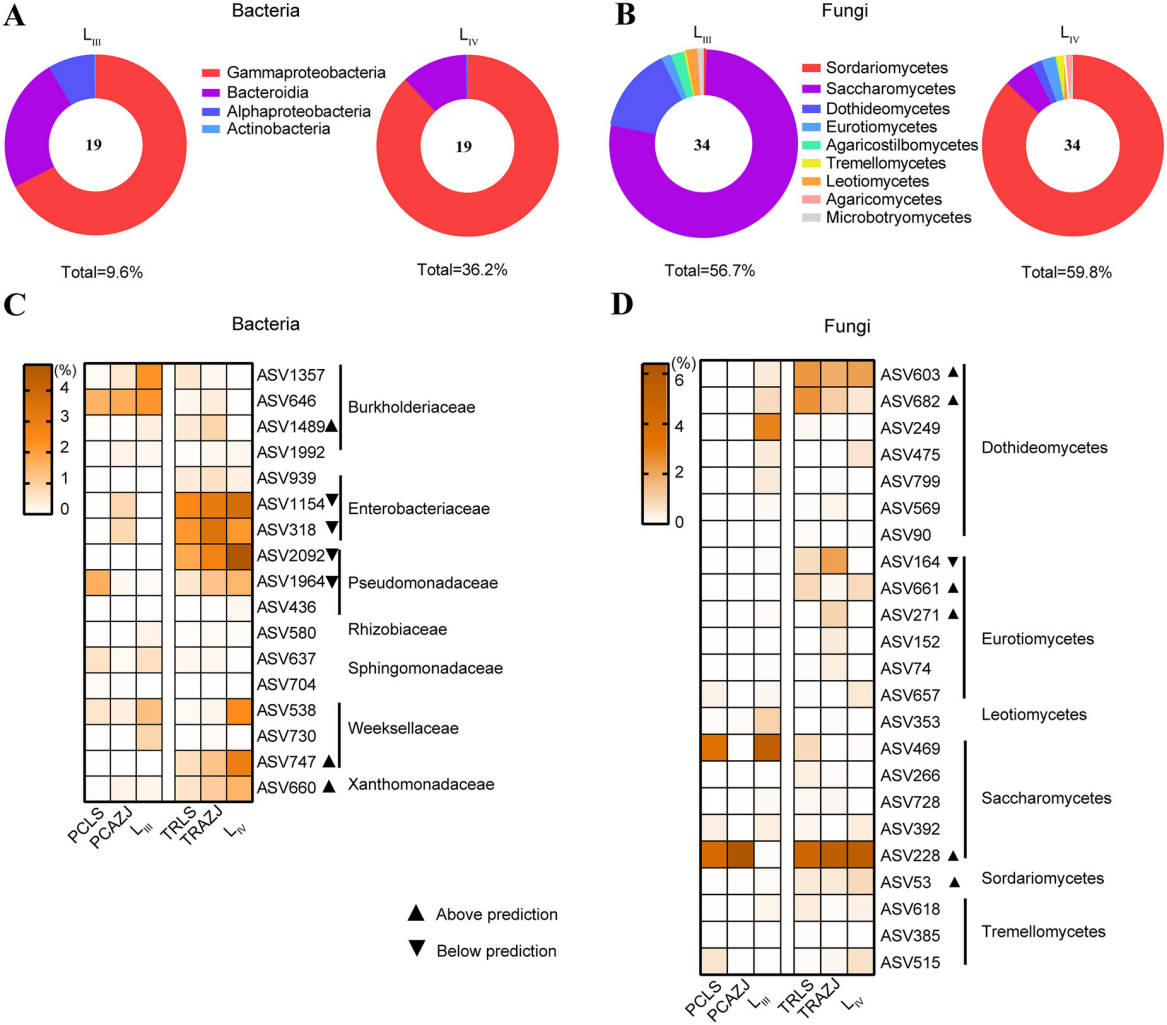
Keystone taxa across the pinewood nematode life cycle. (A and B) Taxonomic composition of the core microbiota taxa at class level across the five sampling sites for L_III_ and L_IV_. (C and D) Heatmap for keystone taxa enriched in L_III_ and L_IV_. The ASVs above or below prediction in the neutral model are marked with triangles. PCLS, pupal chamber samples from Liaoning and Shaanxi; PCAZJ, pupal chamber samples from Anhui, Zhejiang, and Jiangsu.

To identify keystone taxa in each microhabitat of the pinewood nematode life cycle, core microbial taxa were primarily selected from the ASVs appearing in all four microhabitats investigated here (pupal chamber, tracheae, and the third- and fourth-stage pinewood nematode dispersal juveniles). We also identified the widespread ASVs (ASVs present in at least 80% of pupal chamber and tracheae samples, which were divided into two site groups). In total, 19 (bacteria) and 34 (fungi) core ASVs were shared among pupal chambers, tracheae, third-stage dispersal juveniles, and fourth-stage dispersal juveniles. For third-stage dispersal juveniles, the core taxa accounted for 9.6% (bacteria) and 56.7% (fungi) of the total sequences. For fourth-stage dispersal juveniles, the core taxa accounted for 36.2% (bacteria) and 59.8% (fungi) of the total sequences. The dominant bacteria class in both third and fourth-stage dispersal juveniles was Gammaproteobacteria (6.5% and 31.9%). However, the most dominant fungi taxa differed between the two stages, Saccharomycetes (44.0%) being the most common in third-stage dispersal juveniles and Sordariomycetes (49.1%) in fourth-stage dispersal juveniles (Fig. 4A and B, Table S6, S7, S8).

Because vector beetles transmit pinewood nematode to pine trees in their tracheae, these tissues should be the potential source of microbes to be transported to the next pinewood nematode life cycle. Fourth-stage dispersal juveniles were enriched with Pseudomonas ASV2092, Pseudomonas 1964, and ASV1154 (*Enterobacteriaceae*) bacteria as well as with ASV228 (*Ophiostomataceae*), *Cladosporium* ASV603, and *Alternaria* ASV682 fungi (Fig. 4C and D) compared to tracheae. For bacteria, *Stenotrophomonas* ASV660 and *Elizabethkingia* ASV747 were distributed above the predicted in the neutral model and four ASVs (*Pseudomonadaceae* and *Enterobacteriaceae*) were distributed below the prediction. However, *Serratia* ASV318, one of the most widespread ASVs in tracheae, was not increased in fourth-stage dispersal juveniles (Table S6, S7, S8). In contrast, third-stage dispersal juveniles were found to be enriched with ASV646 and ASV1357 (*Burkholderiaceae*) and *Nakazawaea* ASV469 compared to pupal chambers. *Serratia* ASV318 and ASV228 (*Ophiostomataceae*) showed different distribution patterns of the neutral model between northern and southern sites. *Serratia* ASV318 was below the prediction in vector beetle tracheae of southern sites (TRAZJ), but neutrally distributed in northern sites which had the shorter duration of pinewood nematode invasion (Table S5).

## DISCUSSION

Our results showed that bacteria and fungi of dispersal stage pinewood nematodes were derived primarily from the host pine (pupal chambers) and vector beetle (tracheae). This pattern is similar to a plant-soil model that bacterial communities in different parts of the plant were enriched from soils and gradually filtered to the endosphere (7), as well as with the results demonstrating a greater similarity in the microbial community among adjacent trophic levels (10). The two dispersal stages of pinewood nematode, however, differed in microbiota communities: the third-stage dispersal juveniles had a similar amount of fungal and bacterial ASVs compared to the pupal chambers, whereas the fourth-stage dispersal juveniles had lower bacterial and fungal richness and diversity compared to the third-stage dispersal juveniles. In our study, almost all bacteria of the fourth-stage dispersal juveniles were derived from tracheae (>90%), indicating that these dispersal stages entering tracheae may not have their own bacterial community. The higher similarity between the third-stage dispersal juveniles and pupal chamber microbiota compared to the fourth-stage dispersal juveniles is not surprising considering that the third-stage juveniles are collected around pupal chambers, where they aggregate to wait for signals from the vector beetle, after which they molt to the fourth-stage dispersal juveniles and enter the tracheae (25). Differences in microbiota between the two dispersal stages may also rise from their exposure to the surrounding microbiota environment: first, by molting to fourth-stage dispersal juveniles, the larvae likely decrease their microbiota load before entering the vector, and second, because these fourth-stage dispersal juveniles lack functioning mouths, they are less likely to acquire microbiota. Besides these factors, differences in type and number of cuticle proteins between the two dispersal stages of pinewood nematode may also contribute to the observed differences in microbiota composition by affecting their abilities to carry bacteria (22, 39). Similarly, low diversity of microbiota has also been found in dauer juveniles of other nematode species (40), and some studies have shown that entomophilic nematodes do not carry their own bacterial communities during host infestations (38, 41). The reduced microbiota load in the fourth-stage dispersal juveniles may therefore be an adaptation to improve their loading success to the insect vector by reducing the risk of accidental introduction of harmful bacteria that could, for instance, lower the beetle’s fitness or increase the likelihood of triggering the beetle’s immune response. Interestingly, pinewood nematodes can even benefit the vector by reducing bacteria, particularly opportunistically pathogenic taxa, in the beetle’s tracheae (39). However, because in this study we focused on the pinewood nematode stages that are most relevant for the transmission of the pine wilt disease (the two dispersal stages), the question of whether the succession and assembly processes of microbial community differ among propagative and dispersal stages of pinewood nematodes, and whether the low microbiota diversity in the fourth-stage dispersal juveniles is an adaptation to vector-mediated transmission, should be addressed in future studies.

Our results also shed light on the transmission of microbes from the host pine to the vector beetle involved, showing that a large proportion of bacterial and fungal microbiota (40.8%) of tracheae were shared with the pupal chambers and that ~50% of the microbes of the tracheae were derived from pupal chambers. According to previous microbiota studies of pine wilt disease, the dominant organisms in both microhabitats were Gammaproteobacteria, Sordariomycetes, and Saccharomycetes (33–35, 42). These results are also similar to those of other studies showing that the local microhabitat environment is an important source for insect microbiota (4, 43, 44). However, many studies (such as those mentioned above) investigating the transmission route of insect microbiota often use leaf-feeding caterpillars as model organisms, which makes the comprehensive comparison to other insects difficult because caterpillar gut microbiota is mainly derived from the digested food and appears to lack resident gut symbionts (6, 45). Such interspecific comparison maybe especially challenging with bark- and wood-boring beetles like the pinewood nematode’s vector beetle as well as insects feeding on decaying wood such as termites, as these groups of insects often have high gut microbiota diversity and often have resident symbionts (45–48). In addition, most studies have focused only on the gut microbiota composition, whereas tracheae are a semiopen system, and therefore, the microbiota in vector beetle’s trachea may be partially derived from the gut, and partially from host pine (pupal chambers). However, we expected the tracheal microbiota to be stable as adult beetles have a relatively stabilized gut microbiota, whereas the pupal stage has lower bacterial diversity compared to other developmental stages (49, 50), and indeed, we found that trachea selectively acquire some key taxa from the microbial community of the pupal chambers.

Although it is unclear whether pinewood nematode changes the microbiota composition of the tree, it has been previously shown that healthy pine trees have lower bacterial diversity, dominated by cyanobacteria, compared to pinewood nematode invaded pines (34). Interestingly, because plant pests can alter the microbiota of the plants, increasing the plant’s vulnerability to plant diseases, it is possible that nematode infestation also makes the trees susceptible to other plant pathogens (16, 51). However, it is important to note that because our main focus was to investigate the potential microbial transmission route among the key participants of pine wilt disease, our study did not compare microbiota composition of pupal chambers between infected and uninfected pines and could not fully scrutinize to what extent how pine wilt disease complex changes the microbiota composition of the host tree.

In microbiome studies, the use of metacommunity ecology methods has recently gained more popularity (4, 52, 53). We used similar methods (neutral community model) to demonstrate that the microbiota in vector beetle tracheae differed in the community assembly processes among sites differing in the duration of pinewood nematode invasion. We found that the deterministic processes were dominated in the community assembly of tracheae microbiota in both sites, which is similar to patterns found in termites (14). We suggest that the invasion of pinewood nematodes may change the selectivity of the tracheae and therefore affect the tracheae microbiota. Research also found that disease state increases selection for specific taxa (54). Only a few ASVs accounted for the highest cumulative relative abundance and were below the prediction for bacteria, suggesting that these ASVs were selected against by the host or restricted to certain beetle individuals (8, 55). Interestingly, our results also suggest that the beetle trachea is more selective to the bacterial community assembly in sites with a longer duration of pinewood nematode invasion, as indicated by the higher R^2^ value in southern sites in the neutral model. Because diseases may change the selectivity of tissues against or for specific microbiota taxa (54), future investigation of whether pinewood nematode invasion changes the selectivity of the trachea and therefore affects the trachea microbiota is warranted.

Fewer nematodes were recovered from the pupal chamber samples from the southern sites, whereas the bacterial genus *Serratia* ASV318 was below the prediction in vector beetle tracheae in southern sites but neutrally distributed in more recently invaded northern sites. Some *Serratia* species, such as Serratia marcescens, are generally widespread commensal bacteria of insects and other animals but can become opportunistic pathogens. For example, S. marcescens can be lethal if it enters the hemolymph and can successfully evade the immune system of the insect host (39, 56, 57). The combination of higher selectivity of tracheae together with lower nematodes but higher *Serratia* abundances in the sites with the longer duration of pinewood nematode invasion tentatively suggests that microbial composition changes occur during the progress of pine wilt disease, which may have an antagonistic effect on the pinewood nematode-vector beetle disease complex. On the other hand, ophiostomatoid fungi are generally considered as beneficial fungi associated with pinewood nematode and its vector beetle, and we found that *Ophiostomataceae* taxa (ASV228) showed strong host selection in sites with the longer duration of pinewood nematode invasion. This demonstrates further proof that the transmission of Ophiostomatoid fungi from pupal chambers to tracheae is under positive selection by the vector beetle, as shown previously with culture-dependent methods (37), and that the presence of these fungi may balance the potential antagonistic effects caused by increased *Serratia* abundance. In our follow-up study, we have demonstrated that S. marcescens is indeed showed insecticidal and nematicidal against pinewood nematodes and its vector beetles. This bacterium is more abundant in sites with a longer duration of pinewood nematode invasion, and may therefore contribute to the prevalence of pine wilt disease (58). Further investigations of the role and dynamics of potentially harmful *Serratia* bacteria and beneficial Ophiostomatoid fungi for the progress and prevalence of pine wilt disease are therefore warranted.

### Conclusion.

In this study, we investigated the microbiota among different participants involved in pine wilt disease and considered both micro- and macrohabitat factors to estimate the transmission route of the pinewood nematode life cycle. The microbiotas of pinewood nematodes and vector beetles were mainly derived from host pine (pupal chambers), and a few keystone taxa were filtered by tracheae of vector beetles and then enriched in dispersal nematodes. The pupal chamber, which is a shared habitat for both pinewood nematode and the vector beetle, harbored the highest richness of bacteria, whereas the fourth-stage dispersal juveniles had the lowest diversity. The microbial community significantly changed in tracheae, but also shared a large proportion of microbes with the pupal chambers. The bacterial community of fourth-stage dispersal juveniles was mainly derived from tracheae, whereas the nematodes do not alter the diversity of the beetle’s microbiota, indicating a mutualistic interaction. Targeted isolation and functional studies of *Serratia* taxa, negatively selected by vector beetles and being tentatively associated with lower nematode abundance, should be taken in the future to investigate the suitability of these bacteria for pine wilt disease biocontrol.

## MATERIALS AND METHODS

All samples involved in this study were collected from pine trees from two northern locations, Liaoning (LN) and Shaanxi (SX), and three southern locations, Anhui (AH), Zhejiang (ZJ), and Jiangsu (JS), in China in the spring (March to April) during the years 2018 to 2020. In each location, the samples were collected from a 10- to 20-km^2^ area. Each year, at least 30 trees infested with pine wilt disease with a diameter at breast height of 10 to 15 cm, were sampled per location (*Pinus tabuliformis*, and *Pinus koraiensis* in LN, *P. tabuliformis* and *Pinus armandi* in SX, and *Pinus massoniana* in AH, ZJ, and JS). Approximately two or three logs (5- to 10-cm diameter, 20 to 30 cm long) were chosen from each tree. The logs from each location were placed in dark plastic containers and kept at 4°C until used for the collection of pupal chamber samples (2018 to 2019), and third-stage dispersal juveniles (2018 to 2020). The rest of the logs were kept in the glasshouse for collection of vector beetles, trachea extraction, and collection of fourth-stage dispersal juveniles collection (2018 to 2019) (33, 37). Other factors involved in the pinewood nematode life cycle among sampling locations were assessed as described in the supplemental material (Table S1).

### Pupal chamber collection.

Samples of pupal chambers were collected from randomly chosen logs from each location per year. Sawdust from the surface of three to five pupal chambers across multiple logs was scraped using a sterile scalpel and tweezers and pooled as one sample. In total, five to seven samples were chosen from each location per year, resulting in 87 samples across the 2 years (2018 and 2019).

### Vector beetle trachea collection, species identification, and pinewood nematode detection.

The logs not used in the pupal chamber collection were divided by the collection site and kept in the glasshouse to collect emerging adult *Monochamus* sp. vector beetles. The beetles from different sites were identified as *Monochamus alternatus* or *Monochamus saltuarius* and sexed. The cytochrome c oxidase I (COI) gene was used for molecular identification (59). The vector beetles were surface sterilized for 1 min, rinsed with 70% ethanol solution, and rinsed three times with sterile water. Because fourth-stage dispersal juveniles are primarily located in the tracheal system of the vector beetle (33, 60), the beetles were dissected under a CKX53 microscope (Olympus, Tokyo, Japan) for trachea extraction (61). All pupal chambers and trachea samples were examined for pinewood nematode by PCR amplification of the species-specific ITS1 region, and all the primers used are shown in Table S3 (62, 63). In total, we collected and detected 394 tracheae samples of the vector beetles over 2 years (2018, *n* = 207; 2019, *n* = 187). The samples from *M. alternatus* were used to compare the microbiotas of vector beetle tracheae with and without fourth-stage dispersal juveniles by utilizing samples from the same host pine species and similar latitudes (AH, ZJ, and JS).

### Dispersal nematode collection.

From the same logs where the pupal chamber samples were collected, third-stage dispersal juveniles were detected in the tissues surrounding pupal chambers using the Baermann funnel technique (64). After collection, both third-stage dispersal juveniles (from pupal chambers) and fourth-stage dispersal juveniles (from tracheae) larvae were examined under a microscope for morphological confirmation. Two stages of dispersal juveniles were surface sterilized in 3% H_2_O_2_ for 10 min and then rinsed three times with phosphate-buffered saline with Tween 20 (PBST) (33, 65). H_2_O_2_ was used instead of alcohol due to its better sterilization efficiency for nematodes and lower nematicidal effect. The Luria-Bertani (LB) plates that spread on the final washing buffer for 24 h were used to identify the sterilization effect of the nematode cuticle. After that, nematode samples (1,000 to ~2,000 nematodes per sample) were ground (TISSUELYSER-24, Shanghai, China) for DNA extraction. In total, we collected 25 nematode samples across the 3 years.

### Total genomic DNA extraction and Illumina sequencing.

The samples from pupal chambers (~0.1g from each sample) and tracheae were re-suspended in 50 μL of sterile 1× phosphate-buffered saline (PBS). All tissues of the related samples (pupal chambers, tracheae, and third- and fourth-stage dispersal juveniles) were stored at −80°C and used to extract separate genomic DNA extractions. DNA extraction was performed on all samples using the High Pure PCR template preparation kit (Roche Applied Science, Mannheim, Germany) following the manufacturer’s procedures. The chloroplast excluding primers targeting the V5-V7 region of the eubacteria 16S rRNA gene and the fungal nuclear ribosomal internal transcribed spacer region (ITS1) were amplified with barcoded primers 799F-1193R and ITS1F-ITS2R, respectively (66, 67) (Table S3). After quantification by the Quantifluor dsDNA system (Promega, Madison, WI, USA), purified amplicons of bacteria and fungi were pooled in equimolar and sequenced on an Illumina MiSeq PE300 platform (Illumina, San Diego, CA, USA) according to standard protocols by Majorbio Bio-pharm Technology Co., Ltd. (Shanghai, China) (see the supplemental material for details).

### 16S rRNA gene and ITS read processing.

Raw sequences were filtered for quality control by using Majorbio. The paired-end raw data were initially merged by FLASH (version 1.2.7) (68). Sequences with low average quality score (Q < 20), reads lower than 50 bp, primer mismatch of >2, and barcode mismatch of >0 were discarded. Barcodes and primers were removed using “-fastx_truncate” in USEARCH v 10.0 (69). The sequences were processed and analyzed in the Quantitative Insights into Microbial Ecology pipeline version 2020.2 (QIIME2) (70). To avoid potential artifactual sequences, we used the “positive-filtered” output table from Deblur (71) and trimmed paired reads to a size of 170 bp (for fungi) or 370 bp (for bacteria) with Deblur to remove sequencing errors. Taxonomy was assigned to Amplicon sequence variants (ASVs) using the q2-feature-classifier (72). Naïve Bayes classifier was trained by using unite 8.2 dynamic reference sequences (fungi) (http://unite.ut.ee) and silva_132_99_16S (bacteria) (73). ASVs classified as mitochondria or chloroplasts were excluded from further analysis. As an additional quality control, ASVs that could not be assigned to a phylum were removed. However, microbial ASVs with more than 20 reads or those that were present in at least two samples were used. The raw sequencing data have been submitted to the NCBI BioProject under accession number PRJNA720535. A total of 164 fungal samples and 257 bacterial samples passed through the rarefaction step and were used for subsequent analysis. No ASVs were found in the negative controls after standard quality filtering.

### Statistical analyses.

Rarefaction was performed on 2,442 and 10,000 sequences per sample for bacteria and fungi, respectively. Shannon index or the number of observed species was used to estimate alpha diversity. The NMDS was ordinated by unweighted and weighted UniFrac distance matrices for beta diversity analysis (74). To test the effects of different factors on community dissimilarity, PERMANOVA tests or nest PERMANOVA was analyzed by the “adonis” function of the vegan package (75) and QIIME2 packages. A Kruskal-Wallis test performed on Majorbio Cloud Platform (www.majorbio.com) was used to evaluate the alpha diversity and the taxonomical differences among pupal chambers, tracheae, and the dispersal stage nematodes. EdgeR’s generalized linear model (GLM) approach (76) was used for ASVs enriched in tracheae compared to pupal chambers, microbial ASVs with > 100 reads, and ASVs that were present in at least two samples. The enrichment of ASVs was analyzed according to their taxonomy using Manhattan plots. A random forest model was conducted to identify the dominant factor predicting alpha and beta diversity with the “A3” and “rfPermute” R package (77–79).

FEAST (80) based on the Bayesian approach was used to estimate the sources of microbial communities in each microhabitat. In this analysis, samples of tracheae and dispersal nematodes (L_III_ and L_IV_) were set as “sinks”, and samples of pupal chambers were set as ‘source’. The neutral community model (NCM) and the modified stochasticity ratio (MST) were used to determine the potential importance of stochastic processes on community assembly (see Supporting Information for details).

The core microbiota of organisms is considered to drive from evolutionary processes, and filtering effects resulting in selection and enrichment of the microbiota are critical for organism’s performance (81). We considered keystone taxa to consist of widespread, core, and enriched taxa. ASVs that were present in at least 80% of samples in pupal chambers and tracheae (using q2-feature table in QIIME2) were considered widespread taxa, whereas ASVs shared with all samples among host pines, dispersal stages of pinewood nematodes, and tracheae (using Venn diagrams) were considered core taxa. Enriched taxa consisted of ASVs that were enriched in dispersal nematodes compared to pupal chambers and beetle tracheae in different sites (Kruskal–Wallis with FDR adjusted, *P* < 0.05). All figures and graphs were made in R (version 3.6.1) (http://www.r-project.org) and adapted in Adobe Illustrator.

### Data availability.

The data sets generated and/or analyzed during the current study are available in the NCBI SRA repository under BioProject number PRJNA720535. Scripts for computational analysis and corresponding raw data are available at https://github.com/Haokai-Tian/Tian2022_microbiome.git.
