# Supplementary material for: Microhabitat Governs the Microbiota of the Pinewood Nematode and Its Vector Beetle: Implication for the Prevalence of Pine Wilt Disease
Source: Microbiol Spectr. 2022 Jun 27;10(4):e00783-22. doi: 10.1128/spectrum.00783-22 (PMC9430308; doi:10.1128/spectrum.00783-22)
Supplement: Supplemental file 1 — Supplemental material. Download spectrum.00783-22-s0001.pdf, PDF file, 1.3 MB [file spectrum.00783-22-s0001.pdf]

Supporting Materials and Methods

To further investigate the correlation between the micro-and macrohabitat factors and microbiota, we recorded several abiotic and biotic factors, such as the temperature of sampling location and the species of the host pine, and vector beetle (Table S1, Fig. S2). The host plant species were: *Pinus tabuliformis*, *Pinus koraiensis*, *Pinus armandi*, *Pinus massoniana*; the vector species were: *Monochamus alternatus*, *Monochamus saltuarius*. We surveyed the number of PWN surrounding randomly chosen pupal chambers from logs each April from 2018 to 2020. The 20g of wood around each pupal chamber was used to recover PWN by the Baermann funnel. The mean number of PWN around pupal chamber from each location was used to estimate the density of the PWN population (PWN number in Figure S3). The mean number of vector beetles per meter of logs for each site was counted to estimate the density of vector beetle (Vector number in Figure S3) (1, 2). In addition, we calculated the mean values for average temperature and rainfall between March and May during this period based on meteorological data platform of China (http://data.cma.cn/site/index.html), but due to the strong co-linearity of these variables with latitude (Variance inflation factor, VIF >10), we used site in the subsequent analyses to control for potential spatial effect on the microbiota composition (Table S1).

Details on PCR amplification

The DNA quality was detected through the NanoDrop ND-2000 spectrophotometer (Thermo Scientific, Wilmington, DE, USA). The concentration of extracted DNA was

between 35 and 120 ng ml^{-1}. The PCR reaction was performed in triplicate 20 μl of the mixture containing: 10 x buffer, 250 μM dNTPs, 5 μM of each primer (Majorbio, Shanghai, China), 0.2 μL of rTaq DNA Polymerase (TaKaRa, Dalian, China), 10 ng of template and DNA free water. The PCR conditions were as follows: 3 min of a single denaturation step at 95 ºC, 27 cycles of 95 ºC for 30 s, 55 ºC for 30 s, 72 ºC for 45 s, and finished after a final extension at 72ºC for 10 min. The purified PCR products were diluted to 10 ng μl^{-1} as templates for amplification of the second step. All samples were amplified with second-step primers by 13 cycles, other conditions were consistent with the first step. The "blanks" as negative controls were processed in the same procedure to avoid contamination and there was no visible amplification from negative control (no template added). The PCR products were run on a 2% agarose gel and purified with an AxyPrep DNA gel extraction kit (Axygen, Union City, USA) (3, 4).

Analysis of neutral community model and modified stochasticity ratio

The Neutral community model (NCM) was used to predict the relationship between ASV detection frequency and their relative abundance across wider metacommunity (5-8). In general, the model predicts that taxa that are abundant in the metacommunity will be widespread, since they are more likely to disperse by chance among different sampling sites, whereas rare taxa are more likely to be lost in different sites due to ecological drift (8-11). The metacommunity of tracheae samples was separately used the bacterial and fungal datasets from northern and southern sites. The NCM was performed with the R code from Burns et al (2016) and the R^2 values represent the goodness of fit for the NCM (5). Based on the 95% confidence interval of the NCM

predictions, ASVs were separated into three distributions: above prediction, below prediction, and neutral distribution (9, 10, 12). We also used the modified stochasticity ratio (MST) to quantify the relative importance of deterministic and stochastic processes in microbial community assembly (MST value <50%, deterministic-dominance; MST value >50%, stochastic-dominance). The MST analyses were performed with the package of “nst”, based on Bray–Curtis distance (13).

Supporting reference

1. Zhao L, Lu M, Niu H, Fang G, Zhang S, Sun J. 2013. A native fungal symbiont facilitates the prevalence and development of an invasive pathogen-native vector symbiosis. Ecology 94:2817-2826.
2. Zhao L, Zhang X, Wei Y, Zhou J, Zhang W, Qin P, Chinta S, Kong XB, Liu YP, Yu HY, Hu SN, Zou Z, Butcher RA, Sun JH. 2016. Ascarosides coordinate the dispersal of a plant-parasitic nematode with the metamorphosis of its vector beetle. Nat Commun 7:12341.
3. Durán P, Thiergart T, Garrido-Oter R, Agler M, Kemen E, Schulze-Lefert P, Hacquard S. 2018. Microbial Interkingdom Interactions in Roots Promote *Arabidopsis* Survival. Cell 175:973.
4. Zhang J, Liu Y, Zhang N, Hu B, Jin T, Xu H, Qin Y, Yan PX, Zhang XN, Guo XX, Hui J, Cao SY, Wang X, Wang C, Wang H, Qu BY, Fan GY, Yuan LX, Garrido-Oter R, Chu CC, Bai Y. 2019. NRT1.1B is associated with root microbiota composition and nitrogen use in field-grown rice. Nat Biotechnol 37:676-684.
5. Sloan WT, Lunn M, Woodcock S, Head IM, Nee S, Curtis TP. 2006. Quantifying the roles of immigration and chance in shaping prokaryote community structure. Environ Microbiol 8:732-740.
6. Zhou J, Ning D. 2017. Stochastic Community Assembly: Does It Matter in Microbial Ecology? Microbiol Mol Biol Rev 81:e00002-17.

7. Dini-Andreote F, Stegen JC, van Elsas JD, Salles JF. 2015. Disentangling mechanisms that mediate the balance between stochastic and deterministic processes in microbial succession. Proc Natl Acad Sci USA 112:E1326-E1332.
8. Adair KL, Wilson M, Bost A, Douglas AE. 2018. Microbial community assembly in wild populations of the fruit fly *Drosophila melanogaster*. ISME J 12:959-972.
9. Burns AR, Stephens WZ, Stagaman K, Wong S, Rawls JF, Guillemin K, Bohannan BJM. 2016. Contribution of neutral processes to the assembly of gut microbial communities in the zebrafish over host development. ISME J 10:655-664.
10. Wang Y, Wang K, Huang L, Dong P, Wang S, Chen H, Lu Z, Hou D, Zhang D. 2020. Fine-scale succession patterns and assembly mechanisms of bacterial community of *Litopenaeus vannamei* larvae across the developmental cycle. Microbiome 8:106.
11. Nemergut DR, Schmidt SK, Fukami T, O'Neill SP, Bilinski TM, Stanish LF, Knelman JE, Darcy JL, Lynch RC, Wickey P, Ferrenberg S. 2013. Patterns and Processes of Microbial Community Assembly. Microbiol Mol Biol Rev 77:342-356.
12. Chen W, Ren K, Isabwe A, Chen H, Liu M, Yang J. 2019. Stochastic processes shape microeukaryotic community assembly in a subtropical river across wet and dry seasons. Microbiome 7:138.
13. Ning D, Deng Y, Tiedje JM, Zhou J. 2019. A general framework for quantitatively assessing ecological stochasticity. Proc Natl Acad Sci USA 116:16892-16898.

Supplementary figures

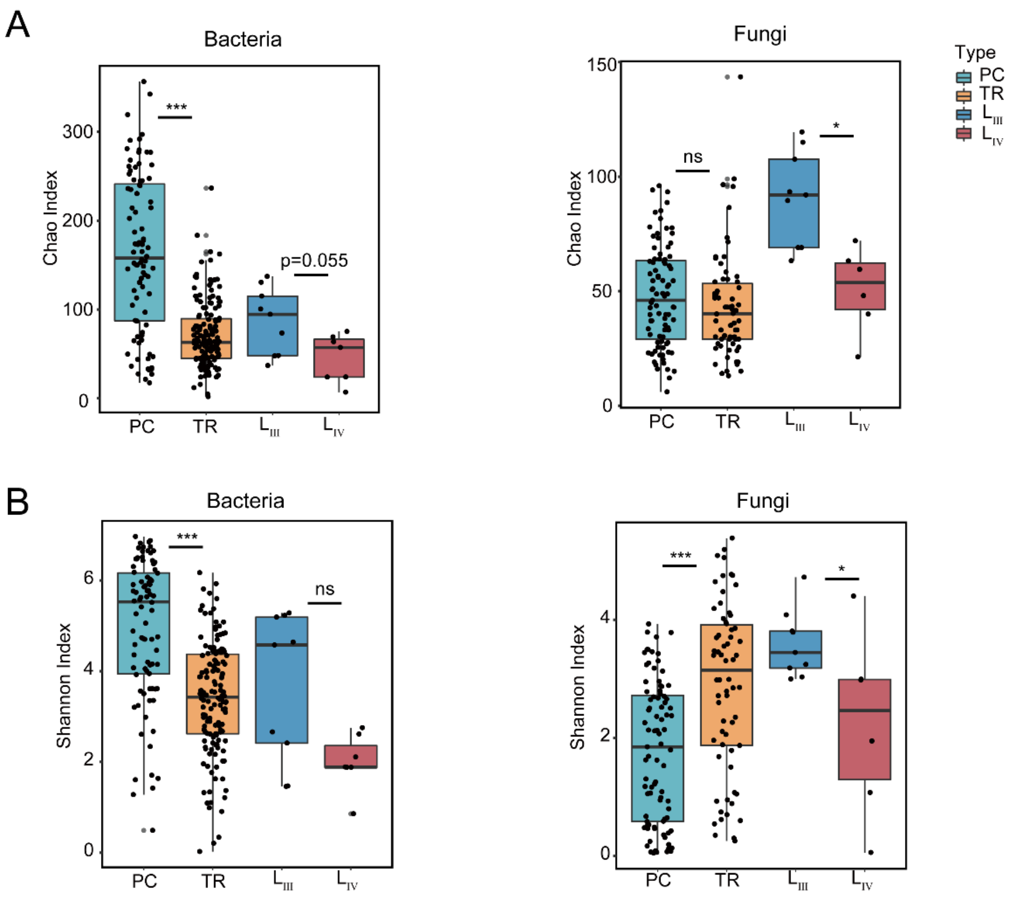

Fig S1 Microbial alpha diversity of different microhabitats. A Chao Index; **B** Shannon Index. The box-and-whisker plots depict the median number of Shannon index in each site and variation is shown in the scatterplot. (**P* < 0.05, ***P* < 0.01, ****P* < 0.001, ns, not significant). PC, pupal chamber; TR, trachea; L_{III}, Third-stage dispersal juveniles; L_{IV}, Fourth-stage dispersal juveniles.

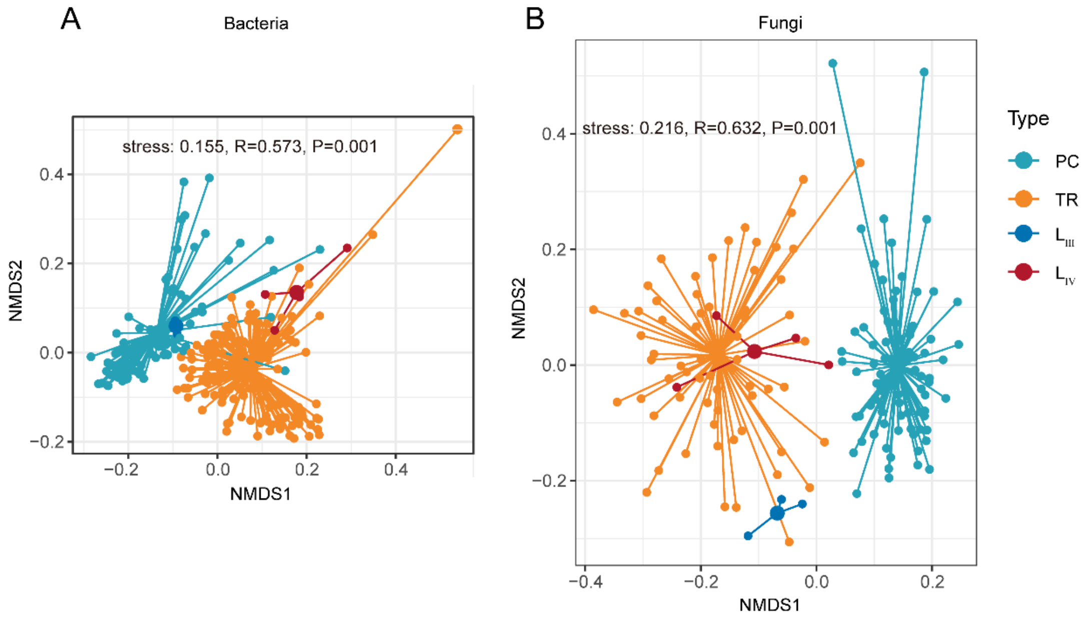

Fig S2 Microbial beta diversity of different microhabitats. The nonmetric multidimensional scaling (NMDS) of bacterial and fungal communities. The clustering was based on Unweighted UniFrac distances. PC, pupal chamber; TR, trachea; L_{III}, Third-stage dispersal juveniles; L_{IV}, Fourth-stage dispersal juveniles.

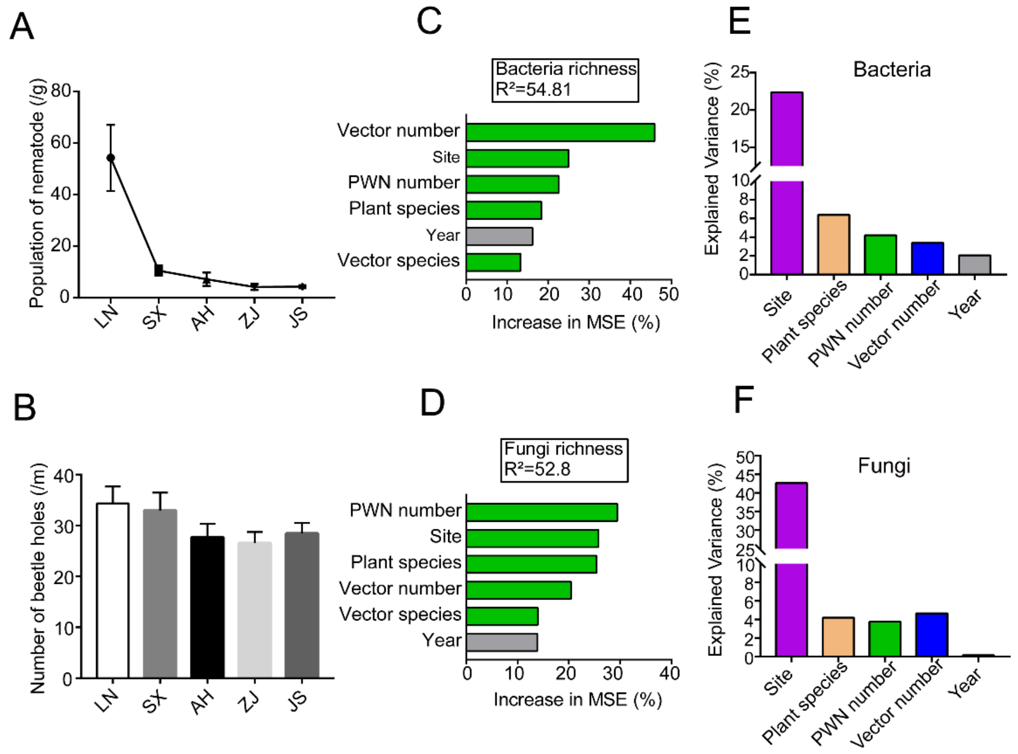

Fig S3 The fraction of the variation in alpha and beta diversity of pupal chamber bacteria and fungi explained by related factors. (A, B) The population density of PWNs and vector beetles in the five sapling locations (2018 and 2019). Bars show mean + SE. (**C, D)** Variable importance estimated by random forest for predicting alpha diversity of PC. (Increased mean square error, %IncMSE). Significant factors are shown in green ($P< 0.05$). **(E, F)** Effect of related factors on microbial community composition of PC. The explained variance (%) was calculated based on PERMANOVA ($P<0.001$). LN: Liaoning; SX: Shaanxi; AH: Anhui; ZJ: Zhejiang; JS: Jiangsu.

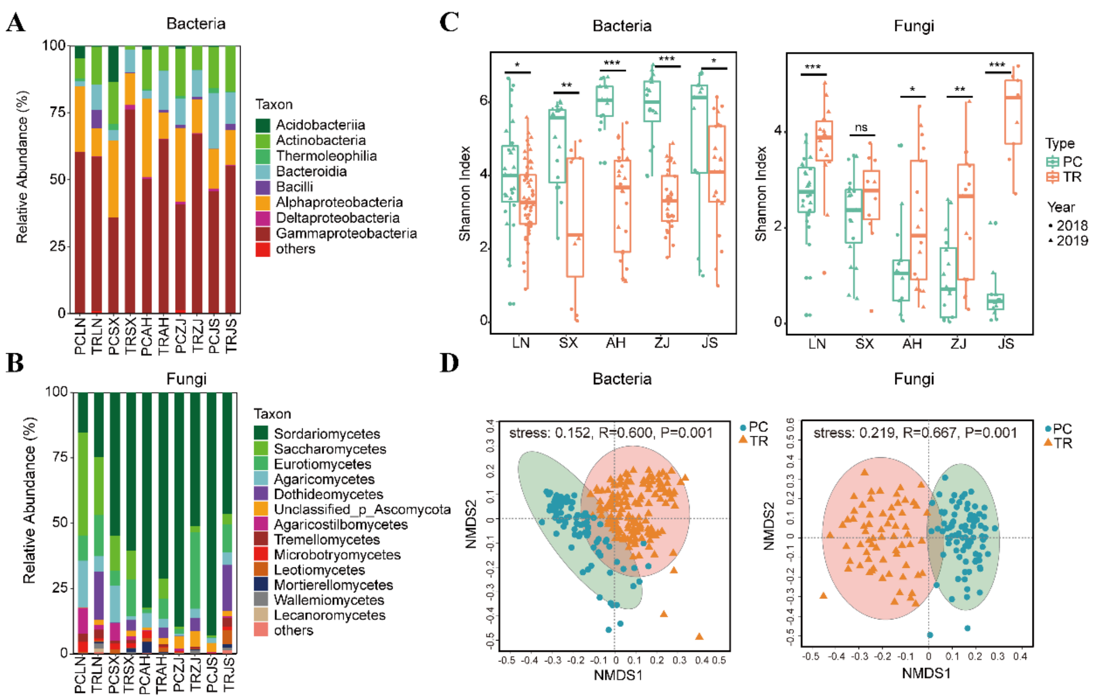

Fig S4 Microbial community structure of pupal chambers and tracheae in all five sites. (A, B) Fungal and bacterial classes at different sites of pupal chambers and tracheae. The different histograms report only taxa with a relative abundance >0.1%. **C** Microbial alpha diversity was measured across all five sites in pupal chamber and tracheae samples based on the Shannon index. Data points corresponding to samples from the two years (circles = 2018, triangles = 2019). Wilcoxon rank-sum test, ns, not significant, * $P < 0.05$, ** $P < 0.01$, *** $P < 0.001$. **D** NMDS of bacterial and fungal communities. The clustering was based on Unweighted UniFrac distances. LN: Liaoning; SX: Shaanxi; AH: Anhui; ZJ: Zhejiang; JS: Jiangsu. PC, pupal chamber; TR, trachea.

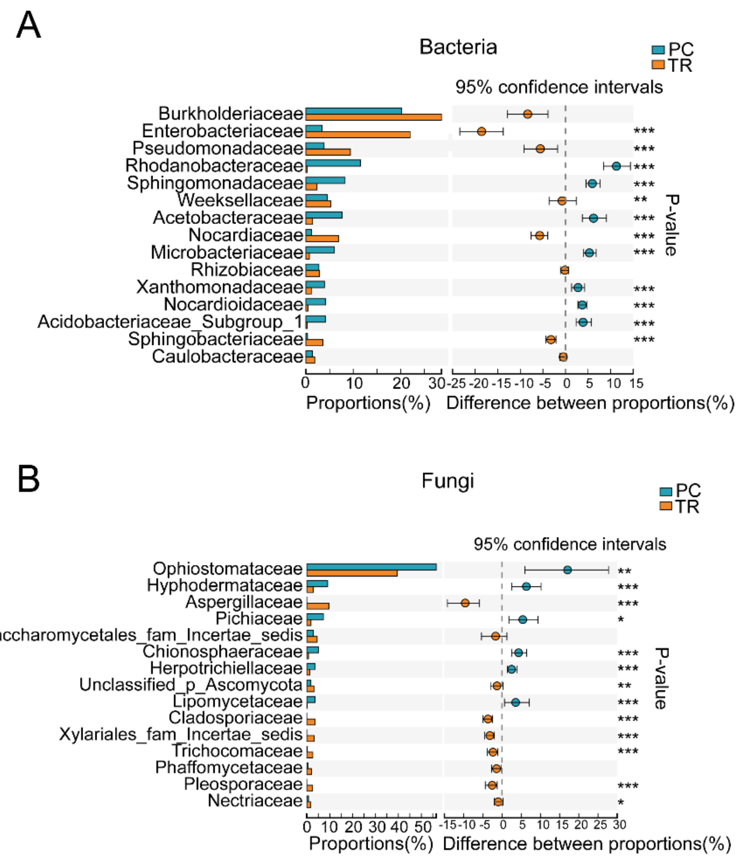

Fig S5 Differential abundance analysis between the pupal chambers and tracheae among the top 15 families. **A** Bacteria; **B** Fungi. (Wilcoxon rank-sum test, FDR < 0.05, **P* < 0.05, ***P* < 0.01, ****P* < 0.001). PC, pupal chamber; TR, trachea.

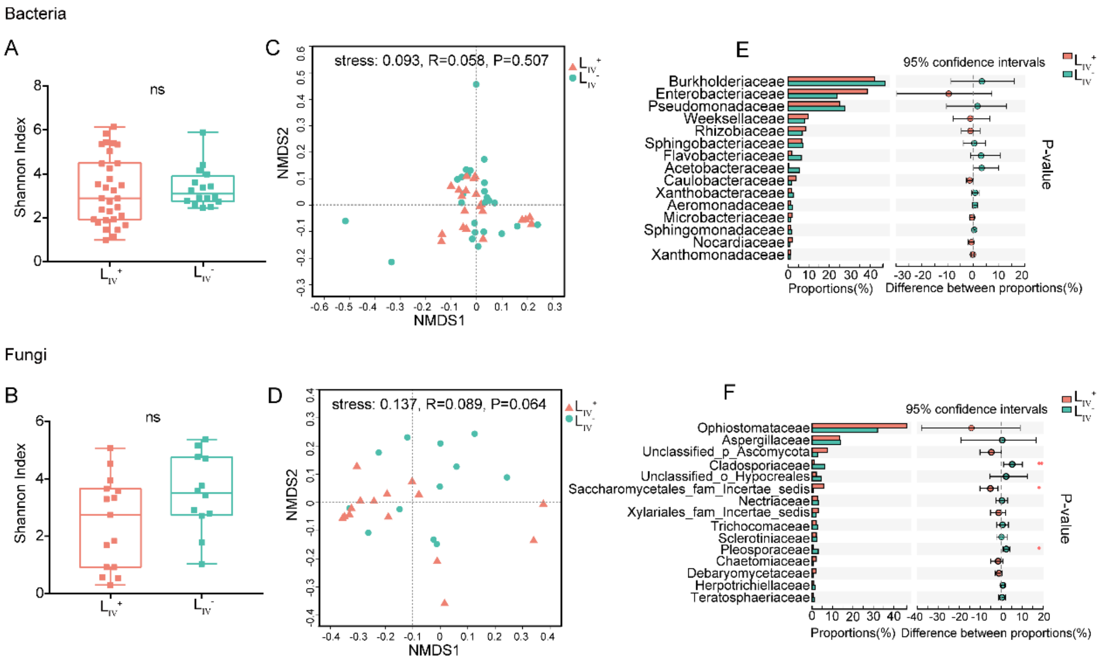

Fig S6 The different members of the microbial community between vector beetles with or without fourth-stage dispersal juveniles. (A, B) Shannon index of the microbiota of tracheae in the three sites (AH, ZJ, JS) involved or uninvolved with L_{IV}. (t-test, ns, not significant) **(C, D)** NMDS of bacterial and fungal communities and grouped by L_{IV}+ and L_{IV}-. The clustering was based on weighted UniFrac similarity. **(E, F)** Comparison of taxa relative abundance (RA) between L_{IV}+ and L_{IV}- samples of tracheae for bacteria and fungi. RA was measured in tracheae across the three southern sites. taxa were aggregated at the family levels. (Wilcoxon rank-sum test, FDR<0.05, **P* < 0.05, ***P* < 0.01, ****P* < 0.001). PC, pupal chamber; TR, trachea; L_{IV}, Fourth-stage dispersal juveniles.

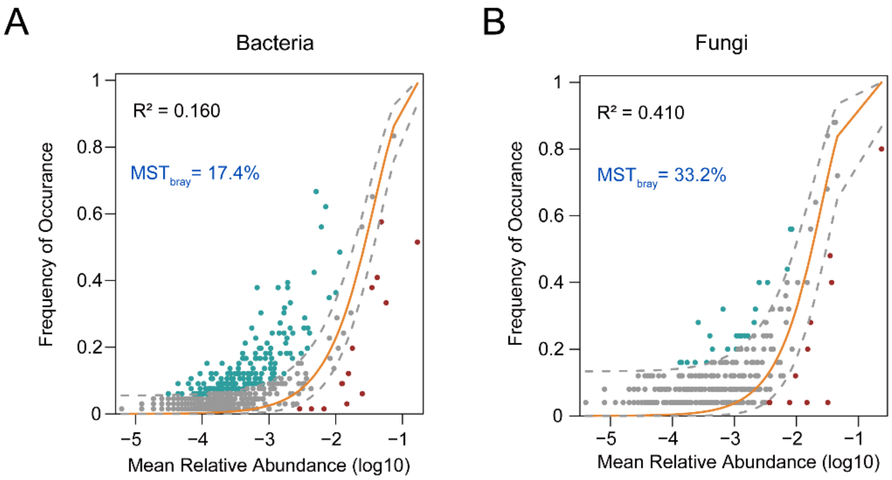

Fig S7 NCM of bacterial and fungal communities among tracheae samples across northern sites (TRLS). TRLS, Trachea samples in Liaoning and Shaanxi.

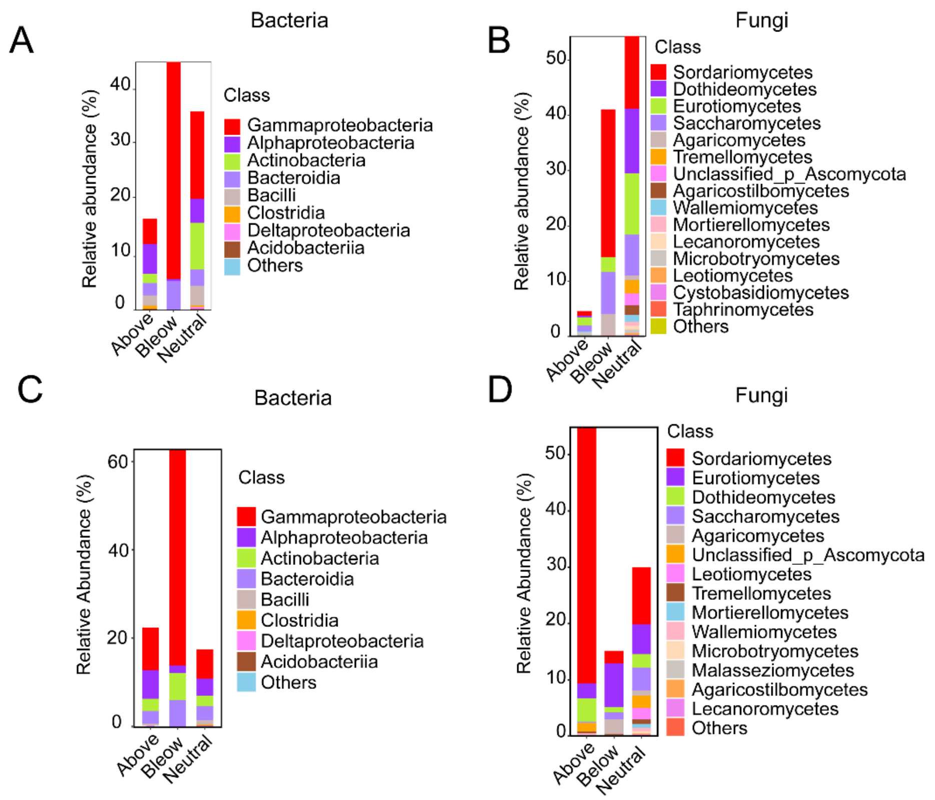

Fig S8 The taxonomic distribution of three categories of ASVs in the neutral model of tracheae in the northern and southern sites. **(A, B)** Vector beetle tracheae of northern sites (TRLS). **(C, D)** Vector beetle tracheae of northern sites (TRAZJ). TRLS, trachea samples in Liaoning and Shaanxi; TRAZJ, trachea samples in Anhui, Zhejiang, and Jiangsu.

Supporting Table legends

Table S1 Summary of collection details.

Table S2 Number of samples collected in this study and samples for data analysis.

Table S3 The primers used in this study.

Table S4 Variable importance estimated by random forest for predicting alpha diversity of pupal chambers and tracheae.

Table S5 The ASVs which were above or below prediction in the neutral model of tracheae microhabitat.

Table S6 Core taxa of bacterial and fungal community among four microhabitats.

Table S7 Widespread ASVs of bacteria inhabiting different host microhabitats.

Table S8 Widespread ASVs of fungi inhabiting different host microhabitats.

Table S1 Summary of collection details.

Site	Province	City	County	Annual average temperature(℃)	Annual average rainfall(mm)	Duration of nematode-vector symbiosis investigation	Latitude	Longitude
LN	Liaoning	Fushun	Fushun	6.1	795.3	2018-2020	41°56′16.844″	124°13′06.227″
			Xinbin			2018-2020	41°58′02.844″	124°24′35.331″
SX	Shaanxi	Shangluo	Zhashui	13.4	741.9	2018-2019	33°34′39.838″	109°16′46.980″
AH	Anhui	Chizhou	Qingyang	16.7	1398.6	2018-2020	30°34′16.901″	117°46′25.203″
ZJ	Zhejiang	Fuyang	Xindeng	16.2	1460	2018-2019	29°58′11.592″	119°43′46.934″
			Dongqiao			2018-2019	30°4′34.933″	119°35′05.539″
JS	Jiangsu	Changzhou	Tianmuhu	15.8	1120.6	2018-2020	31°20′02.753″	119°26′28.817″
			Shangxin			2018-2020	31°31′43.982″	119°15′53.510″

Table S2 Number of samples collected in this study and samples for data analysis.

Samples we collected in this study:

Compartment	Smaples number		
	2018	2019	2020
Pupal Chamber	40	47	
Trachea	207	187	
Nematode	14	2	9

Samples which sequenced successfully for data analysis:

Compartment	Fungi (n=164)			Bacteria (n=257)		
	2018	2019	2020	2018	2019	2020
Pupal Chamber	40	46		39	47	
Trachea	30	29		90	58	
Nematode	9	2	8	12	2	9

Table S3 The primers used in this study.

**Primer**	**Sequence (5'-3')**	**Length (bp)**	**Target**	**Reference**
799F	AACMGGATTAGATACCCKG	593	Bacteria 16S rRNA gene region for Illumina sequencing	Bulgarelli *et al*, (2015)
1192R	ACGTCATCCCCACCTTCC			
799F	AACMGGATTAGATACCCKG	394		
1193R	ACGTCATCCCCACCTTCC			
ITS1F	CTTGGTCATTTAGAGGAAGTAA	Around 300	Fungi ITS gene region for Illumina sequencing	Admas *et al*, (2013)
ITS2R	GCTGCGTTCTTCATCGATGC			
Bx-F	CGATGATGCGATTGGTGACT	767	Species identification	FILIPIAK *et al*, (2017)
Bx-R	CCCAACTCAGGTGGTTTCAA		Species identification	
8F	AGAGTTTGATCATGGCTCAG	1500	Species identification	Egert *et al*, (2005)
1492R	TACGGTTACCTTGTTACGACTT		Species identification	
C1-J-2183a	CAACAYTTATTTTGATTTTTTGG	800-1000	Species identification	Cardoso *et al,* (2012)
TL2-N-3014	TCCAATGCACTAATCTGCCATATTA		Species identification	

Table S4 Variable importance estimated by random forest for predicting alpha diversity of pupal chambers and tracheae.

Host types	Effects	Bacteria			Fungi		
		%IncMSE	*P*	R^2	%IncMSE	*P*	R^2
Pupal chamber	Vector number	45.96	0.01	54.81	*20.44*	0.02	52.80
	Site	24.97	0.01		25.79	0.02	
	PWN number	22.56	0.02		29.46	0.02	
	Plant species	18.40	0.01		25.38	0.02	
	Year	16.21	0.16		13.88	0.16	
	Vector species	13.28	0.01		13.97	0.02	
Trachea	Site	3.55	0.31	2.21	28.37	0.02	25.49
	PWN number	-1.70	0.56		24.41	0.02	
	Vector number	31.42	0.02		21.99	0.08	
	Vector species	3.08	0.23		11.52	0.06	
	Plant species	4.87	0.16		11.00	0.18	
	Year	11.25	0.02		10.87	0.26	

Table S5 The ASVs which were above or below prediction in neutral model of tracheae microhabitat.

Bacteria: Below prediction

**ASV_ID**	**Phylum**	**Class**	**Order**	***Family***	***Genus***
ASV318*	Proteobacteria	Gammaproteobacteria	Enterobacteriales	*Enterobacteriaceae*	*Serratia*
ASV1154	Proteobacteria	Gammaproteobacteria	Enterobacteriales	*Enterobacteriaceae*	*Unclassified*
ASV1118	Actinobacteria	Actinobacteria	Corynebacteriales	*Nocardiaceae*	*Rhodococcus*
ASV2092*	Proteobacteria	Gammaproteobacteria	Pseudomonadales	*Pseudomonadaceae*	*Pseudomonas*
ASV1891	Proteobacteria	Gammaproteobacteria	Betaproteobacteriales	*Burkholderiaceae*	*Cupriavidus*
ASV977	Proteobacteria	Gammaproteobacteria	Betaproteobacteriales	*Burkholderiaceae*	*Unclassified*
ASV2017	Proteobacteria	Gammaproteobacteria	Betaproteobacteriales	*Burkholderiaceae*	*Unclassified*
ASV1253	Bacteroidetes	Bacteroidia	Sphingobacteriales	*Sphingobacteriaceae*	*Sphingobacterium*
ASV862	Proteobacteria	Gammaproteobacteria	Enterobacteriales	*Enterobacteriaceae*	*Enterobacter*
ASV1964*	Proteobacteria	Gammaproteobacteria	Pseudomonadales	*Pseudomonadaceae*	*Pseudomonas*
ASV2074	Bacteroidetes	Bacteroidia	Flavobacteriales	*Flavobacteriaceae*	*Flavobacterium*
ASV62	Proteobacteria	Gammaproteobacteria	Enterobacteriales	*Enterobacteriaceae*	*Unclassified*
ASV1098	Proteobacteria	Gammaproteobacteria	Pseudomonadales	*Pseudomonadaceae*	*Pseudomonas*
ASV777	Bacteroidetes	Bacteroidia	Flavobacteriales	*Weeksellaceae*	*Chryseobacterium*
ASV673	Proteobacteria	Gammaproteobacteria	Enterobacteriales	*Enterobacteriaceae*	*Unclassified*
ASV2111	Proteobacteria	Alphaproteobacteria	Acetobacterales	*Acetobacteraceae*	*Acetobacter*
ASV1084	Proteobacteria	Gammaproteobacteria	Pseudomonadales	*Pseudomonadaceae*	*Pseudomonas*
ASV858	Proteobacteria	Gammaproteobacteria	Enterobacteriales	*Enterobacteriaceae*	*Unclassified*
ASV1058	Proteobacteria	Gammaproteobacteria	Enterobacteriales	*Enterobacteriaceae*	*Unclassified*

ASV1094	Proteobacteria	Gammaproteobacteria	Betaproteobacteriales	*Burkholderiaceae*	*Burkholderia-Caballeronia-Paraburkholderia*
ASV1046	Proteobacteria	Alphaproteobacteria	Rhizobiales	*Rhizobiaceae*	*Allorhizobium-Neorhizobium-Pararhizobium-Rhizobium*
ASV1972	Proteobacteria	Gammaproteobacteria	Enterobacteriales	*Enterobacteriaceae*	*Unclassified*
ASV1849	Proteobacteria	Gammaproteobacteria	Enterobacteriales	*Enterobacteriaceae*	*Unclassified*
ASV720	Bacteroidetes	Bacteroidia	Flavobacteriales	*Weeksellaceae*	*Chryseobacterium*
ASV806	Bacteroidetes	Bacteroidia	Flavobacteriales	*Weeksellaceae*	*Chryseobacterium*
ASV1123	Bacteroidetes	Bacteroidia	Flavobacteriales	*Weeksellaceae*	*Empedobacter*

Fungi: Above prediction

ASV_ID	Phylum	Class	Order	Family	Genus
ASV32	Ascomycota	Sordariomycetes	Hypocreales	*Ophiocordycipitaceae*	*Hirsutella*
ASV53*	Ascomycota	Sordariomycetes	Hypocreales	*Hypocreaceae*	*Trichoderma*
ASV62	Basidiomycota	Malasseziomycetes	Malasseziales	*unidentified*	*unidentified*
ASV76	Ascomycota	Eurotiomycetes	Eurotiales	*Aspergillaceae*	*Penicillium*
ASV96	Basidiomycota	Malasseziomycetes	Malasseziales	*Malasseziaceae*	*Malassezia*
ASV98	Ascomycota	Unclassified_p_Ascomycota	Unclassified	*Unclassified*	*Unclassified*
ASV152	Ascomycota	Eurotiomycetes	Eurotiales	*Aspergillaceae*	*Penicillium*
ASV178	Basidiomycota	Tremellomycetes	Trichosporonales	*Trichosporonaceae*	*Cutaneotrichosporon*
ASV207	Ascomycota	Eurotiomycetes	Chaetothyriales	*unidentified*	*unidentified*
ASV208	Ascomycota	Eurotiomycetes	Eurotiales	*Unclassified*	*Unclassified*
ASV228*	Ascomycota	Sordariomycetes	Ophiostomatales	*Ophiostomataceae*	*Unclassified*
ASV249	Ascomycota	Dothideomycetes	Pleosporales	*Didymellaceae*	*Unclassified*
ASV271*	Ascomycota	Eurotiomycetes	Eurotiales	*Aspergillaceae*	*Penicillium*
ASV316	Ascomycota	Dothideomycetes	Capnodiales	*Mycosphaerellaceae*	*Mycosphaerella*
ASV371	Ascomycota	Eurotiomycetes	Eurotiales	*Aspergillaceae*	*Aspergillus*
ASV374	Ascomycota	Saccharomycetes	Saccharomycetales	*Saccharomycetaceae*	*Saccharomyces*
ASV383	Ascomycota	Eurotiomycetes	Eurotiales	*Aspergillaceae*	*Aspergillus*
ASV396	Ascomycota	Eurotiomycetes	Chaetothyriales	*Herpotrichiellaceae*	*Unclassified*
ASV426	Ascomycota	Eurotiomycetes	Chaetothyriales	*Herpotrichiellaceae*	*Unclassified*
ASV457	Ascomycota	Saccharomycetes	Saccharomycetales	*Pichiaceae*	*Nakazawaea*
ASV461	Basidiomycota	Wallemiomycetes	Wallemiales	*Wallemiaceae*	*Wallemia*
ASV530	Ascomycota	Eurotiomycetes	Eurotiales	*Aspergillaceae*	*Penicillium*

ASV557	Ascomycota	Eurotiomycetes	Eurotiales	*Aspergillaceae*	*Aspergillus*
ASV569	Ascomycota	Dothideomycetes	Capnodiales	*Cladosporiaceae*	*Cladosporium*
ASV603*	Ascomycota	Dothideomycetes	Capnodiales	*Cladosporiaceae*	*Cladosporium*
ASV607	Basidiomycota	Agaricomycetes	Agaricales	*Schizophyllaceae*	*Schizophyllum*
ASV612	Ascomycota	Sordariomycetes	Hypocreales	*Nectriaceae*	*Xenoacremonium*
ASV618	Basidiomycota	Tremellomycetes	Filobasidiales	*Filobasidiaceae*	*Filobasidium*
ASV661	Ascomycota	Eurotiomycetes	Eurotiales	*Aspergillaceae*	*Penicillium*
ASV663	Ascomycota	Dothideomycetes	Capnodiales	*Unclassified*	*Unclassified*
ASV682*	Ascomycota	Dothideomycetes	Pleosporales	*Pleosporaceae*	*Alternaria*
ASV706	Ascomycota	Dothideomycetes	Pleosporales	*Didymellaceae*	*Unclassified*
ASV721	Ascomycota	Eurotiomycetes	Eurotiales	*Aspergillaceae*	*Penicillium*
ASV741	Ascomycota	Saccharomycetes	Saccharomycetales	*Saccharomycetaceae*	*Saccharomyces*
ASV752	Ascomycota	Saccharomycetes	Saccharomycetales	*Saccharomycetaceae*	*Saccharomyces*
ASV754	Ascomycota	Sordariomycetes	Hypocreales	*Nectriaceae*	*Fusarium*
ASV755	Ascomycota	Eurotiomycetes	Chaetothyriales	*Herpotrichiellaceae*	*Capronia*
ASV794	Ascomycota	Saccharomycetes	Saccharomycetales	*Saccharomycetaceae*	*Saccharomyces*
ASV804	Ascomycota	Sordariomycetes	Hypocreales	*Nectriaceae*	*Fusarium*
ASV808	Ascomycota	Sordariomycetes	Togniniales	*Togniniaceae*	*Phaeoacremonium*
ASV824	Ascomycota	Eurotiomycetes	Chaetothyriales	*Herpotrichiellaceae*	*Fonsecaea*
ASV846	Basidiomycota	Wallemiomycetes	Wallemiales	*Wallemiaceae*	*Wallemia*

The ASVs which were above or below prediction in neutral model of tracheae microhabitat across three southern sites.

*The ASVs which showed different distribution pattern in neutral model of northern sites.

Table S6 Core taxa of bacterial and fungal community among four microhabitats.

Bacteria: N=19

ASVs	Class	Order	Family	Genus	Avg. abundance in pupal chamber (%)	Avg. abundance in trachea (%)	Avg. abundance in L_{III} (%)	Avg. abundance in L_{IV} (%)
ASV1118	Actinobacteria	Corynebacteriales	*Nocardiaceae*	*Rhodococcus*	3.46E-01	6.63E+00	2.24E-02	2.18E-02
ASV637	Alphaproteobacteria	Sphingomonadales	*Sphingomonadaceae*	*Novosphingobium*	2.96E-01	1.24E-01	5.64E-01	2.35E-02
ASV1574	Alphaproteobacteria	Rhizobiales	*Xanthobacteraceae*	*Rhodopseudomonas*	3.00E-01	4.70E-02	5.08E-02	1.09E-02
ASV580	Alphaproteobacteria	Rhizobiales	*Rhizobiaceae*	*Allorhizobium-Neorhizobium-Pararhizobium-Rhizobium*	1.83E-03	5.42E-02	1.84E-01	5.28E-02
ASV704	Alphaproteobacteria	Sphingomonadales	*Sphingomonadaceae*	*Sphingobium*	5.67E-02	1.46E-03	5.59E-03	1.41E-02
ASV538	Bacteroidia	Flavobacteriales	*Weeksellaceae*	*Chryseobacterium*	3.46E-01	1.30E-01	1.51E+00	4.21E+00
ASV730	Bacteroidia	Flavobacteriales	*Weeksellaceae*	*Chryseobacterium*	5.31E-03	3.68E-02	8.08E-01	1.09E-02
ASV2092	Gammaproteobacteria	Pseudomonadales	*Pseudomonadaceae*	*Pseudomonas*	8.60E-04	4.36E+00	1.01E-02	2.74E+01
ASV1357	Gammaproteobacteria	Betaproteobacteriales	*Burkholderiaceae*	*Burkholderia-Caballeronia-Paraburkholderia*	2.20E-01	2.28E-01	3.71E+00	9.41E-03
ASV1964	Gammaproteobacteria	Pseudomonadales	*Pseudomonadaceae*	*Pseudomonas*	1.18E+00	8.96E-01	6.07E-02	1.85E+00
ASV601	Gammaproteobacteria	Enterobacteriales	*Enterobacteriaceae*	*Serratia*	1.32E+00	3.82E-01	1.97E+00	1.06E-01
ASV660	Gammaproteobacteria	Xanthomonadales	*Xanthomonadaceae*	*Stenotrophomonas*	1.09E-01	7.84E-01	1.49E-01	1.91E+00
ASV38	Gammaproteobacteria	Pseudomonadales	*Pseudomonadaceae*	*Pseudomonas*	6.47E-01	1.89E-01	1.11E-01	6.12E-02
ASV2040	Gammaproteobacteria	Enterobacteriales	*Enterobacteriaceae*	*Unclassified*	9.10E-02	8.61E-01	3.14E-02	2.24E-02
ASV1489	Gammaproteobacteria	Betaproteobacteriales	*Burkholderiaceae*	*Delftia*	2.66E-02	6.16E-01	2.47E-01	1.88E-02

ASV939	Gammaproteobacteria	Enterobacteriales	*Enterobacteriaceae*	*Unclassified*	6.41E-04	4.18E-01	3.04E-02	2.30E-01
ASV1001	Gammaproteobacteria	Betaproteobacteriales	*Burkholderiaceae*	*Variovorax*	3.83E-01	2.50E-02	7.45E-02	9.41E-03
ASV1992	Gammaproteobacteria	Betaproteobacteriales	*Burkholderiaceae*	*Comamonas*	9.74E-02	7.61E-02	9.90E-02	1.24E-01
ASV436	Gammaproteobacteria	Pseudomonadales	*Pseudomonadaceae*	*Pseudomonas*	6.60E-03	1.58E-02	5.59E-03	1.22E-01
Total					5.43	15.87	9.64	36.23

Fungi: N=34

**ASVs**	**Class**	**Order**	**Family**	**Genus**	**Avg. abundance in PC (%)**	**Avg. abundance in TR (%)**	**Avg. abundance in L_{III} (%)**	**Avg. abundance in L_{IV} (%)**
ASV469	Saccharomycetes	Saccharomycetales	*Pichiaceae*	*Nakazawaea*	5.45E+00	3.23E-01	4.18E+01	6.67E-02
ASV249	Dothideomycetes	Pleosporales	*Didymellaceae*	*Unclassified*	3.00E-04	7.37E-02	6.22E+00	3.65E-02
ASV620	Saccharomycetes	Saccharomycetales	*Lipomycetaceae*	*Lipomyces*	7.86E-01	8.62E-02	1.64E+00	6.20E-02
ASV471	Agaricostilbomycetes	Agaricostilbales	*Chionosphaeraceae*	*Chionosphaera*	4.69E+00	7.50E-01	1.28E+00	9.29E-02
ASV353	Leotiomycetes	Helotiales	*Unclassified*	*Unclassified*	5.61E-02	3.30E-02	1.03E+00	2.84E-03
ASV682	Dothideomycetes	Pleosporales	*Pleosporaceae*	*Alternaria*	6.72E-03	2.49E+00	7.49E-01	4.74E-01
ASV5	Microbotryomycetes	Microbotryomycetes_ord_Incertae_sedis	*Chrysozymaceae*	*Trigonosporomyces*	8.57E-01	9.06E-02	5.53E-01	1.78E-03
ASV799	Dothideomycetes	Pleosporales	*Didymellaceae*	*Didymella*	1.55E-04	3.78E-02	4.26E-01	9.97E-04
ASV755	Eurotiomycetes	Chaetothyriales	*Herpotrichiellaceae*	*Capronia*	1.08E+00	2.70E-01	3.88E-01	1.34E-01
ASV603	Dothideomycetes	Capnodiales	*Cladosporiaceae*	*Cladosporium*	5.54E-03	3.23E+00	3.81E-01	3.47E+00
ASV475	Dothideomycetes	Dothideales	*Aureobasidiaceae*	*Unclassified*	9.06E-05	1.19E-02	3.61E-01	5.11E-01
ASV392	Saccharomycetes	Saccharomycetales	*Saccharomycetales_fam_Incertae_sedis*	*Kuraishia*	1.65E-01	5.24E-02	3.03E-01	3.48E-01

ASV621	Eurotiomycetes	Chaetothyriales	*Herpotrichiellaceae*	*Unclassified*	1.32E-01	4.04E-02	2.43E-01	9.93E-03
ASV618	Tremellomycetes	Filobasidiales	*Filobasidiaceae*	*Filobasidium*	5.23E-03	1.79E-01	1.79E-01	2.73E-01
ASV657	Eurotiomycetes	Chaetothyriales	*Herpotrichiellaceae*	*Capronia*	1.13E-01	1.49E-02	1.38E-01	4.30E-01
ASV21	Agaricomycetes	Polyporales	*Hyphodermataceae*	*Hyphoderma*	8.75E+00	2.71E+00	1.17E-01	8.11E-01
ASV728	Saccharomycetes	Saccharomycetales	*Debaryomycetaceae*	*Debaryomyces*	6.56E-04	1.45E-01	1.15E-01	2.67E-03
ASV569	Dothideomycetes	Capnodiales	*Cladosporiaceae*	*Cladosporium*	2.40E-04	1.38E-01	1.14E-01	4.46E-03
ASV727	Sordariomycetes	Ophiostomatales	*Ophiostomataceae*	*Ophiostoma*	1.22E+00	7.80E-01	1.09E-01	1.21E-01
ASV53	Sordariomycetes	Hypocreales	*Hypocreaceae*	*Trichoderma*	2.53E-02	4.07E-01	8.80E-02	8.37E-01
ASV271	Eurotiomycetes	Eurotiales	*Aspergillaceae*	*Penicillium*	2.74E-04	6.09E-01	6.96E-02	1.22E-02
ASV324	Saccharomycetes	Saccharomycetales	*Saccharomycetales_fam_Incertae_sedis*	*Unclassified*	6.00E-01	1.46E+00	6.52E-02	2.53E+00
ASV293	Sordariomycetes	Calosphaeriales	*Pleurostomataceae*	*Pleurostoma*	9.33E-02	6.64E-01	4.54E-02	4.66E-01
ASV228	Sordariomycetes	Ophiostomatales	*Ophiostomataceae*	*Unclassified*	4.95E+01	3.62E+01	3.58E-02	4.74E+01
ASV74	Eurotiomycetes	Eurotiales	*Trichocomaceae*	*Talaromyces*	4.96E-04	1.92E-01	3.45E-02	3.99E-03
ASV164	Eurotiomycetes	Eurotiales	*Aspergillaceae*	*Penicillium*	9.75E-04	2.40E+00	3.32E-02	1.83E-02
ASV661	Eurotiomycetes	Eurotiales	*Aspergillaceae*	*Penicillium*	2.41E-04	4.08E-01	2.95E-02	7.62E-01
ASV789	Sordariomycetes	Ophiostomatales	*Ophiostomataceae*	*Sporothrix*	3.25E+00	1.22E+00	2.41E-02	2.65E-01
ASV266	Saccharomycetes	Saccharomycetales	*Debaryomycetaceae*	*Meyerozyma*	3.93E-03	1.58E-01	2.16E-02	1.35E-02
ASV515	Tremellomycetes	Tremellales	*Tremellales_fam_Incertae_sedis*	*Cuniculitrema*	2.90E-01	1.10E-01	2.13E-02	6.05E-01
ASV90	Dothideomycetes	Dothideales	*Unclassified*	*Unclassified*	2.34E-04	3.52E-02	5.31E-03	3.48E-02
ASV385	Tremellomycetes	Tremellales	*Bulleraceae*	*Pseudotremella*	1.24E-03	1.61E-02	5.31E-03	1.22E-03
ASV152	Eurotiomycetes	Eurotiales	*Aspergillaceae*	*Penicillium*	7.83E-04	2.27E-01	4.61E-03	1.43E-02
ASV555	Sordariomycetes	Diaporthales	*Valsaceae*	*Cytospora*	2.16E-02	9.07E-03	2.66E-03	3.55E-03
Total					77.11	55.57	56.66	59.77

Table S7 Widespread ASVs of bacteria inhabiting in different host microhabitat which dominated in pupal chambers and tracheae.

**ASVs**	**Family**	**Genus**	**PCAZJ-mean (%)**	**TRAZJ-mean (%)**	**L_{III}-mean (%)**	**L_{IV}-mean (%)**	**PCLS-mean (%)**	**TRLS-mean (%)**	**Corrected pvalue**
ASV127	*Xanthomonadaceae*	*Pseudoxanthomonas*	1.86E+00	2.13E+00	1.17E-01	8.11E-01	1.49E+01	3.64E+00	4.06E+01
ASV298	*Burkholderiaceae*	*Unclassified*	2.83E-01	8.64E-01	0.00E+00	3.95E+00	9.79E-02	2.24E-01	3.09E+01
ASV557	*Burkholderiaceae*	*Variovorax*	1.34E+00	1.47E+00	0.00E+00	2.41E-02	4.75E-04	1.29E-01	8.14E+01
ASV646	*Burkholderiaceae*	*Burkholderia-Caballeronia-Paraburkholderia*	1.05E-03	2.43E-02	0.00E+00	7.70E-01	2.57E+00	5.61E-01	9.81E+01
ASV914	*Rhodanobacteraceae*	*Dyella*	4.27E-02	6.71E-01	0.00E+00	6.08E-03	1.18E-02	2.08E-01	5.18E+01
ASV1001	*Burkholderiaceae*	*Variovorax*	8.32E+01	4.39E+01	3.58E-02	4.74E+01	1.77E+01	2.37E+01	7.75E+01
ASV1094	*Burkholderiaceae*	*Burkholderia-Caballeronia-Paraburkholderia*	2.29E+00	1.98E+00	0.00E+00	3.47E-02	7.77E-02	6.87E-01	3.71E+01
ASV1191	*Acetobacteraceae*	*Acidisoma*	5.05E-01	1.20E+00	0.00E+00	4.20E-01	6.33E-01	1.50E+00	1.22E+01
ASV1451	*Acidobacteriaceae (Subgroup 1)*	*Edaphobacter*	5.25E-01	1.67E-01	0.00E+00	1.53E-01	1.68E-01	2.03E-02	2.09E+01
ASV1465	*Acetobacteraceae*	*Roseococcus*	4.07E-01	0.00E+00	0.00E+00	4.26E-02	1.91E-03	0.00E+00	9.59E+01
ASV1568	*Burkholderiaceae*	*Pandoraea*	5.93E-02	6.91E-02	0.00E+00	6.13E-01	1.30E+00	3.16E+00	5.40E+01
ASV1673	*Xanthomonadaceae*	*Pseudoxanthomonas*	6.40E-04	2.43E-02	4.18E+01	6.67E-02	1.03E+01	8.13E-01	9.70E+01
ASV1717	*Burkholderiaceae*	*Burkholderia-Caballeronia-Paraburkholderia*	5.24E-01	1.75E-01	1.28E+00	9.29E-02	8.43E+00	1.69E+00	8.70E+01
ASV1787	*67-14*	*Unclassified*	4.17E-01	9.25E-01	0.00E+00	5.78E-02	0.00E+00	7.97E-03	6.77E+01
ASV143	*Moraxellaceae*	*Acinetobacter*	1.91E-01	5.47E-03	0.00E+00	7.30E-02	5.34E-01	6.76E-03	5.40E+01
ASV318	*Enterobacteriaceae*	*Serratia*	3.99E-01	1.74E-01	3.88E-01	1.34E-01	1.71E+00	4.27E-01	5.87E+01
ASV388	*Weeksellaceae*	*Chryseobacterium*	7.89E-03	7.05E-02	2.41E-02	2.65E-01	6.15E+00	3.11E+00	8.86E+01
ASV652	*Burkholderiaceae*	*Delftia*	1.46E-01	2.30E-01	1.78E-01	0.00E+00	8.71E-01	3.28E-01	4.91E+01

ASV747	*Weeksellaceae*	*Elizabethkingia*	8.32E+01	4.39E+01	3.58E-02	4.74E+01	1.77E+01	2.37E+01	7.75E+01
ASV1118	*Nocardiaceae*	*Rhodococcus*	0.00E+00	2.57E+00	4.59E-02	5.67E-01	0.00E+00	4.00E+00	9.99E+01
ASV1154	*Enterobacteriaceae*	*Unclassified*	1.34E+00	1.47E+00	0.00E+00	2.41E-02	4.75E-04	1.29E-01	8.14E+01
ASV2017	*Burkholderiaceae*	*Unclassified*	4.55E-03	2.62E+00	3.81E-01	3.47E+00	6.33E-03	4.22E+00	1.07E+02

Bacterial taxa which present in more than 80% of samples in pupal chamber and tracheae

PC, pupal chamber; TR, trachea; L_{III}, Third-stage dispersal juveniles; L_{IV}, Fourth-stage dispersal juveniles; PCLS, Pupal chamber of Liaoning and Shaanxi; PCAZJ, Pupal chamber samples in Anhui, Zhejiang, and Jiangsu.

Table S8 Widespread ASVs of fungi inhabiting in different host microhabitat which dominated in pupal chambers and tracheae.

ASVs	Family	Genus	PCAZJ-mean (%)	TRAZJ-mean (%)	L_{III}-mean (%)	L_{IV}-mean (%)	PCLS-mean (%)	TRLS-mean (%)	Corrected pvalue
ASV21	*Hyphodermataceae*	*Hyphoderma*	1.86E+00	2.13E+00	1.17E-01	8.11E-01	1.49E+01	3.64E+00	4.06E+01
ASV95	*Saccharomycetales_fam_Incertae_sedis*	*Kuraishia*	2.83E-01	8.64E-01	0.00E+00	3.95E+00	9.79E-02	2.24E-01	3.09E+01
ASV98	*Unclassified*	*Unclassified*	1.34E+00	1.47E+00	0.00E+00	2.41E-02	4.75E-04	1.29E-01	8.14E+01
ASV118	*Herpotrichiellaceae*	*Capronia*	1.05E-03	2.43E-02	0.00E+00	7.70E-01	2.57E+00	5.61E-01	9.81E+01
ASV162	*Ophiostomataceae*	*Sporothrix*	4.27E-02	6.71E-01	0.00E+00	6.08E-03	1.18E-02	2.08E-01	5.18E+01
ASV228	*Ophiostomataceae*	*Unclassified*	8.32E+01	4.39E+01	3.58E-02	4.74E+01	1.77E+01	2.37E+01	7.75E+01
ASV394	*Unclassified*	*Unclassified*	2.29E+00	1.98E+00	0.00E+00	3.47E-02	7.77E-02	6.87E-01	3.71E+01
ASV418	*Phaffomycetaceae*	*Cyberlindnera*	5.05E-01	1.20E+00	0.00E+00	4.20E-01	6.33E-01	1.50E+00	1.22E+01
ASV426	*Herpotrichiellaceae*	*Unclassified*	5.25E-01	1.67E-01	0.00E+00	1.53E-01	1.68E-01	2.03E-02	2.09E+01
ASV443	*Microbotryomycetes_fam_Incertae_sedis*	*Colacogloea*	4.07E-01	0.00E+00	0.00E+00	4.26E-02	1.91E-03	0.00E+00	9.59E+01
ASV457	*Pichiaceae*	*Nakazawaea*	5.93E-02	6.91E-02	0.00E+00	6.13E-01	1.30E+00	3.16E+00	5.40E+01
ASV469	*Pichiaceae*	*Nakazawaea*	6.40E-04	2.43E-02	4.18E+01	6.67E-02	1.03E+01	8.13E-01	9.70E+01
ASV471	*Chionosphaeraceae*	*Chionosphaera*	5.24E-01	1.75E-01	1.28E+00	9.29E-02	8.43E+00	1.69E+00	8.70E+01
ASV612	*Nectriaceae*	*Xenoacremonium*	4.17E-01	9.25E-01	0.00E+00	5.78E-02	0.00E+00	7.97E-03	6.77E+01
ASV750	*Chrysozymaceae*	*Hamamotoa*	1.91E-01	5.47E-03	0.00E+00	7.30E-02	5.34E-01	6.76E-03	5.40E+01
ASV755	*Herpotrichiellaceae*	*Capronia*	3.99E-01	1.74E-01	3.88E-01	1.34E-01	1.71E+00	4.27E-01	5.87E+01
ASV789	*Ophiostomataceae*	*Sporothrix*	7.89E-03	7.05E-02	2.41E-02	2.65E-01	6.15E+00	3.11E+00	8.86E+01
ASV833	*Microbotryomycetes_fam_Incertae_sedis*	*Colacogloea*	1.46E-01	2.30E-01	1.78E-01	0.00E+00	8.71E-01	3.28E-01	4.91E+01
ASV645	*Xylariales_fam_Incertae_sedis*	*Phialemoniopsis*	0.00E+00	2.57E+00	4.59E-02	5.67E-01	0.00E+00	4.00E+00	9.99E+01
ASV603	*Cladosporiaceae*	*Cladosporium*	4.55E-03	2.62E+00	3.81E-01	3.47E+00	6.33E-03	4.22E+00	1.07E+02
ASV682	*Pleosporaceae*	*Alternaria*	4.89E-03	1.19E+00	7.49E-01	4.74E-01	8.25E-03	4.62E+00	7.99E+01

Fungal taxa which present in more than 80% of samples in pupal chambers and tracheae.

PC, pupal chamber; TR, trachea; L_{III}, Third-stage dispersal juveniles; L_{IV}, Fourth-stage dispersal juveniles; PCLS, Pupal chamber of Liaoning and Shaanxi; PCAZJ, Pupal chamber samples in Anhui, Zhejiang, and Jiangsu.
